# Supplementary material for: Combining data and theory for derivable scientific discovery with AI-Descartes
Source: Nat Commun. 2023 Apr 12;14:1777. doi: 10.1038/s41467-023-37236-y (PMC10097814; doi:10.1038/s41467-023-37236-y)
Supplement: Supplementary file 1 — Supplementary Information [file 41467_2023_37236_MOESM1_ESM.pdf]

Supplementary Information

Combining Data and Theory for Derivable Scientific Discovery with AI-Descartes

Authors: C. Cornelio, S. Dash, V. Austel, T. R. Josephson, J. Goncalves, K. Clarkson, N. Megiddo, B. El Khadir, L. Horesh.

Supplementary Note 1 - Datasets

Supplementary Note 1.1 - Kepler's third law of planetary motion

		Normalization factors		
	Original	Solar	Exoplanet	Binary Stars
p	$[s]$	$1000 \cdot 24 \cdot 60 \cdot 60$	$1000 \cdot 24 \cdot 60 \cdot 60$	$365 \cdot 24 \cdot 60 \cdot 60$ [y]
m_1	$[kg]$	$1.9885 \cdot 10^{30}$	$1.9885 \cdot 10^{30}$	$1.9885 \cdot 10^{30}$
m_2	$[kg]$	$5.972 \cdot 10^{24}$	$1.898 \cdot 10^{27}$	$1.9885 \cdot 10^{30}$
d	$[m]$	$1.496 \cdot 10^{11}$ $[au]$	$1.496 \cdot 10^{11}$ $[au]$	$1.496 \cdot 10^{11}$ $[au]$

Supplementary Table 1. Units of measurement and normalization factors for Kepler data

We use three different datasets [1–3], provided in Supplementary Table 2. The first has eight data points corresponding to eight planets of the solar system. The second has 20 data points consisting of all eight data points from the first dataset, and, in addition, data points corresponding to some exoplanets in the Trappist-1 and the GJ 667 systems. The third consists of data for four binary stars systems [3]. All three datasets consist of real measurements of four variables: the distance d between two bodies (a star and an orbiting planet in the first two datasets and binary stars in the third dataset), the masses m_1 and m_2 of the two bodies, and the orbital period p, which is our target variable. We normalized the data (e.g., masses of planets and stars) to reduce errors that can arise due to processing large numbers in our system. Supplementary Table 1 gives the original unit of measurement and the normalization factors for each dataset. Each star mass is given as a multiple of the mass of the sun, hence the sun mass equals 1. In the first dataset, each planetary mass is given as a multiple of Earth's mass, whereas in the second dataset each planetary mass is given relative to Jupiter's mass. The distance d is given in astronomical units [au]. The period p is given as days/1000 (first two datasets) or years (third dataset).

Supplementary Note 1.2 - Relativistic time dilation

We report in Supplementary Table 3 the data used in the work of Chou et al. [4, Figure 2]. The first column gives the velocity of a moving clock relative to another stationary clock, and the second column gives the relative change in clock rates (i.e., it gives the clock rate, or frequency, of the moving clock minus the clock rate of the stationary clock divided by the clock rate of the stationary clock) scaled by 10^{15}.

We next list and describe the axioms given as input to the reasoning module for this problem (see Supplementary Table 4). The period dt_0 of a "light clock" is defined as the time for light (at velocity c) to travel between two stationary mirrors separated by distance d (axiom R1 below). The period dt of a similar pair of mirrors moving with velocity v (axiom R2), is the time taken for light to bounce between the two mirrors, but in this case, while traveling the distance L (calculated via the Pythagorean theorem in axiom R3). The observed change in clock frequency due to motion, $df = f - f_0$, (axiom R6) is related to periods dt_0 and dt using definitions of frequency (axioms A4 and R5). The second column of the previous table gives values for df/f_0 (after scaling by 10^{15}). Here all variables are positive, and the speed of light is taken to be 3×10^8 meters per second (axioms R7-R8). In Supplementary Figure 1, the dashed gray lines represent lengths, the solid yellow lines represent the direction of travel for light (if vertical or diagonal) and the solid black lines represent the direction of motion of the light source (horizontal).

Solar				Exoplanet				Binary stars			
m_1	m_2	d	p	m_1	m_2	d	p	m_1	m_2	d	p
1.0	0.0553	0.3870	0.0880	1.0	0.000174	0.3870	0.0880	0.54	0.50	107.270	1089.0
1.0	0.815	0.7233	0.2247	1.0	0.00256	0.7233	0.2247	1.33	1.41	38.235	143.1
1.0	1.0	1.0	0.3652	1.0	0.00315	1.0	0.3652	0.88	0.82	113.769	930.0
1.0	0.107	1.5234	0.6870	1.0	0.000338	1.5234	0.6870	3.06	1.97	131.352	675.5
1.0	317.83	5.2045	4.331	1.0	1.0	5.2045	4.331				
1.0	95.16	9.5822	10.747	1.0	0.299	9.5822	10.747				
1.0	14.54	19.2012	30.589	1.0	0.0457	19.2012	30.589				
1.0	17.15	30.0475	59.800	1.0	0.0540	30.0475	59.800				
				0.33	0.018	0.0505	0.0072004				
				0.33	0.012	0.125	0.02814				
				0.33	0.008	0.213	0.06224				
				0.33	0.008	0.156	0.039026				
				0.33	0.014	0.549	0.2562				
				0.08	0.0027	0.0111	0.0015109				
				0.08	0.0043	0.0152	0.0024218				
				0.08	0.0013	0.0214	0.0040496				
				0.08	0.002	0.0282	0.0060996				
				0.08	0.0021	0.0371	0.0092067				
				0.08	0.0042	0.0451	0.0123529				
				0.08	0.086	0.063	0.018767				

Supplementary Table 2. Kepler data

Supplementary Note 1.3 - Langmuir's adsorption equation

In Supplementary Table 5 we provide two datasets, one taken from Langmuir's original paper [5, Table IX], and the other from the work of Sun et al. [6, Table 1]. Each dataset gives the measured loading q at different values of pressure p at a fixed temperature. We note that different scales for pressure and loading are used in these two datasets.

For some experiments (marked with the label "extra point") we added the extra point $p = q = 0$* that modulates the first constraint C1 from $\mathscr{K}$.

Thermodynamic constraints We consider background knowledge in the form of a list of desired properties of the relation between p and q, available for adsorption thermodynamics. Axioms for single-component adsorption are more plausible when they satisfy certain thermodynamic constraints $\mathscr{K}$:

C1. $f(0) = 0$

C2. $(\forall p > 0)\ (f(p) > 0)$

C3. $(\forall p > 0)\ (f'(p) \geq 0)$

C4. $0 < \lim_{p \to 0} f'(p) < \infty$

C5. $0 < \lim_{p \to \infty} f(p) < \infty$

C1 requires that at zero pressure, zero molecules may be adsorbed, and C2 requires that only positive loadings are feasible. C3 requires the isotherm increase monotonically with pressure, which holds for all single-component adsorption systems. C4 requires the slope of the adsorption isotherm in the limit of zero pressure (the adsorption second virial coefficient) to be positive and finite [7]. C5 requires the loading to be finite in the limit of infinite pressure, thus imposing a saturation capacity for the material. These five constraints are satisfied by Langmuir and

*In the implementation we used the approximation $p = q = 0.001$ as our SR solver deals with expressions of the form p^t which is not always defined for $p = 0$, $t < 0$.

Velocity (m/s)	Time dilation (10^{-15})
0.55	-0.018
4.10	-0.21
8.60	-0.43
14.84	-1.54
22.18	-2.92
29.65	-4.82
36.22	-7.36

Supplementary Table 3. Time dilation data

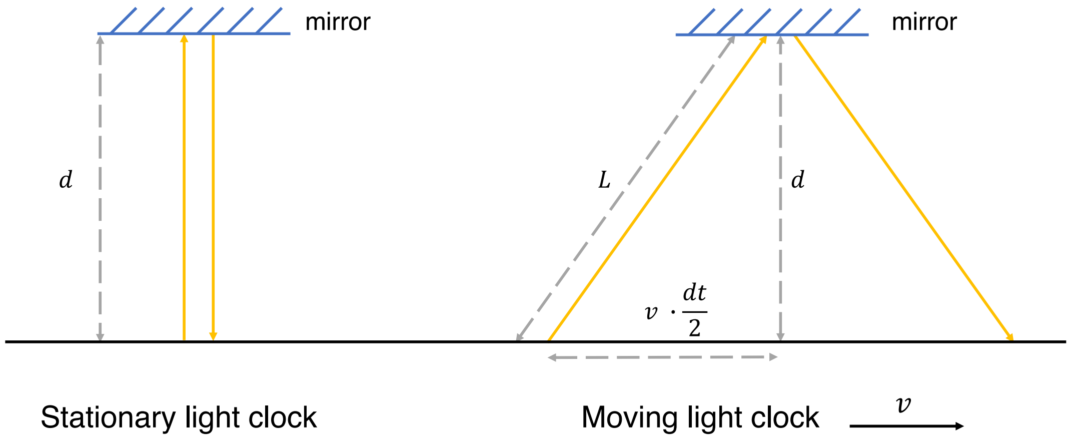

Supplementary Figure 1. Depiction of moving light clock

multi-site Langmuir models, but some popular models in the literature violate these to various degrees. For example, Freundlich and Sips formulae violate constraint C4 – a well-known issue critiqued by Talu and Myers [7]. The BET isotherm violates C2 and C3 because of its singularity at the vapor pressure of the fluid p_{vap}; this could be resolved by instead enforcing positivity and monotonicity from $0 < p < p_{\text{vap}}$.

Supplementary Note 2 - Symbolic regression

A symbolic regression scheme consists of a space of valid mathematical expressions composable from a basic list of operators (we assume these to be unary or binary), and a mechanism for exploring the space. Each valid mathematical expression can be represented by an expression tree, i.e., a rooted binary tree where each non-leaf node has an associated binary or unary operator ($+$, $-$, $\times$, $\sqrt{}$, log, etc.), and each leaf node has an associated constant or independent variable. An example of an expression tree for the expression

$$mx^2\omega^2 + \frac{\omega}{mx} \tag{1}$$

is presented in Supplementary Figure 2 (full expression tree).

Symbolic regression is often solved with genetic programming (GP). The `Eureqa` package [8], based on the work of Schmidt and Lipson [9], is a state-of-the-art GP-based solver. Another popular solver is `gplearn` [10]. Such solvers search the space of expressions using genetic algorithms. Models generated by GP often suffer from poor accuracy [11] and lengthy descriptions.

The symbolic regression problem can be formulated in various ways as a Mixed-Integer Nonlinear-Programming (MINLP) problem (e.g., see [12–15]). The MINLP problem is solved to global optimality using an off-the-shelf

Relativistic axioms		Newtonian axioms	
R1.	$dt_0 = 2d/c$	R1.	$dt_0 = 2d/c$
R2.	$dt = 2L/c$	R2'.	$dt = 2L/\sqrt{v^2+c^2}$
R3.	$L^2 = d^2 + (v\,dt/2)^2$	R3.	$L^2 = d^2 + (v\,dt/2)^2$
R4.	$f_0 = 1/dt_0$	R4.	$f_0 = 1/dt_0$
R5.	$f = 1/dt$	R5.	$f = 1/dt$
R6.	$df = f - f_0$	R6.	$df = f - f_0$
R7.	$d > 0, v > 0$	R7.	$d > 0, v > 0$
R8.	$c = 3 \cdot 10^8$	R8.	$c = 3 \cdot 10^8$

Supplementary Table 4. Axioms for time dilation. The first column give axioms for relativistic time dilation. An alternate set of axioms for "Newtonian behavior" are given in the second column.

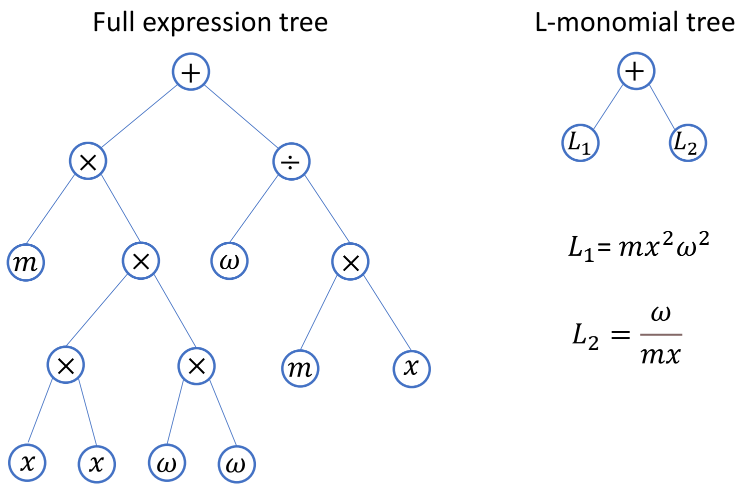

Supplementary Figure 2. Expression tree for the Expression 1. On the left, expressed with full arithmetic notation, and on the right, as a more compact tree of L-monomials.

Langmuir [5, Table IX]		Sun et al. [6, Table 1]			
p	q	p	q	p	q
2.7	30.6	0.07	0.695	12.06	1.371
3.7	36.3	0.11	0.752	17.26	1.469
5.2	43.7	0.20	0.797	27.56	1.535
8.0	52.7	0.31	0.825	41.42	1.577
12.8	60.6	0.56	0.860	55.20	1.602
17.3	71.2	0.80	0.882	68.95	1.619
25.8	82.2	1.07	0.904	86.17	1.632
45.0	90.2	1.46	0.923		
83.0	98.6	3.51	0.976		
122.0	104.0	6.96	1.212		

Supplementary Table 5. Langmuir data

MINLP solver such as `BARON` [16], `COUENNE` or `SCIP` [17]. These solvers solve problems of the form

$$\text{Minimize} \quad f(\mathbf{x},\mathbf{y}) \tag{2}$$
$$\text{s.t.} \quad g(\mathbf{x},\mathbf{y}) \leq \mathbf{b} \tag{3}$$
$$\mathbf{x} \in \mathbb{R}^m, \mathbf{y} \in \mathbb{Z}^m \tag{4}$$

where $\mathbf{x}$ is a vector of m continuous variables, $\mathbf{y}$ is a vector of n discrete variables, $f(\mathbf{x},\mathbf{y})$ is a real-valued function that can be composed using a finite list of operators such as $+,-,\times,/,\exp(\cdot)$ etc. (this list will vary with each solver), and $g(\mathbf{x},\mathbf{y})$ is a vector-valued function created using the same operators. These solvers use various convex-relaxation schemes to obtain lower bounds on the objective-function value, and utilize these bounds in branch-and-bound schemes to obtain globally optimal solutions.

This approach produces a globally-optimal mathematical expression along with a certificate of optimality, while avoiding an exhaustive search of the solution space. Another advantage is that it directly produces correct (within a tolerance) real-valued constants; most other methods use specialized algorithms to refine constants (e.g., [18]) and cannot guarantee global optimality.

In these MINLPs, the set of valid binary expression trees is specified by a set of constraints over discrete and continuous variables. The discrete variables of the formulation are used to define the structure of the expression tree including the assignment of operators to non-leaf nodes and whether a leaf node is assigned a constant or a specific independent variable. The continuous variables are used for the undetermined constants, and also to evaluate the resulting symbolic expression for specific numerical values associated with individual data points. The objective functions vary from *accuracy* (measured as sum of squared deviations) to *model complexity*. The MINLP formulations broadly have the following form:

$$\begin{aligned}
\text{Minimize} \quad & \mathscr{C}(f_\theta) && \text{complexity}\\
\text{s.t.} \quad & \theta \in \mathscr{T} && \text{grammar}\\
& \mathbf{v}_i = f_\theta(\mathbf{X}^{(i)}), \ \forall i \in I && \text{prediction}\\
& \mathscr{D}(f_\theta(\mathbf{X}), \mathbf{Y}) \leq \varepsilon && \text{error}
\end{aligned}$$

where f_θ represents an expression tree defined by the structural and continuous decision variables collectively designated as θ; $\mathscr{T}$ is the universe of valid expression trees; $\mathscr{C}$ measures the description complexity; $\mathscr{D}$ measures error of the predicted values $\mathbf{v}$; $\mathbf{Y}$ is the vector of observations for input vectors $\{\mathbf{X}^{(i)}\}_{i \in I}$.

Supplementary Note 2.1 - System Description

We implemented a symbolic regression system based on a novel mixed-integer nonlinear programming formulation. We first describe the basics of our system (without dimensional analysis), and then later explain how we incorporate dimensional analysis.

We take as input a list of operators (for this discussion, assume the input operators are $+,-,\times,/$, and $\sqrt{}$), an upper bound d on the tree depth, an upper bound k on the number of constants, and a domain for the constants $[-\Omega,\Omega]$. In Supplementary Figure 2, there is a single constant (distinct from 1) with value $1/4$ in the expression tree and in the L-monomial tree. In the prior work on globally optimal symbolic regression, the MINLP formulations typically have discrete variables that (1) determine the placement of the operators in nodes of the expression tree and (2) the mapping of independent variables to leaf nodes, and (3) determine whether to map an independent variable or a constant to a leaf node.

In our formulation, we simply do not have discrete variables for (1). We explicitly enumerate all possible assignments of the operators in expression trees up to depth d. More precisely, if a "partial" expression tree is one where the leaf nodes are undetermined, but the operator assignments to non-leaf nodes are determined, then we enumerate all possible partial expression trees up to a certain depth. Our assignment of variables/constants to leaf nodes is also different from prior work. Let $x_1,\dots,x_n$ be all the independent variables. Instead of assuming that each leaf node is either a constant h or one of $x_1,\dots,x_n$ as in the prior work, we assume each leaf node is a one-term multivariable Laurent polynomial of the form

$$hx_1^{a_1}x_2^{a_2}\cdots x_n^{a_n}, \tag{5}$$

where $a_1,\dots,a_n$ are (undetermined) integers (for computational efficiency, we limit these integers to lie in the range $[-\delta,\delta]$ for some input constant δ), and h represents an (undetermined) constant in the final expression. We refer to an expression of the type (5) as an *L-monomial*. In other words, rather than assigning a single variable or constant to a leaf node, we potentially assign both variables and constants, and also multiple variables (with positive or negative powers to a leaf node). We call the resulting trees *generalized expression trees*, and *gentrees* for convenience.

Each gentree T (with depth d) corresponds to a symbolic expression: each non-leaf node with height 1 corresponds to the expression formed by applying the operator at the node to the expressions (i.e., L-monomials) in the children nodes, and non-leaf nodes at greater heights are handled in the same manner in order of height. The only gentree with depth 0 corresponds to the L-monomial L_1, whereas the gentrees of depth 1 correspond to the expressions $\sqrt{L_1}$, L_1+L_2, $L_1\times L_2$, L_1/L_2 and L_1-L_2, respectively, where L_1 and L_2 are L-monomials.

Supplementary Note 2.2 - Pruning the list of gentrees

We try to reduce the number of relevant partial expression trees/gentrees by removing a number of "redundant" trees using the fact that the set of nonzero (the constant h in 5 is nonzero) L-monomials is closed under multiplication and division. Notice that both $L_1\times L_2$ and L_1/L_2 are L-monomials and thus can be represented by a single expression L_1. In other words, if there is a symbolic expression f of the form $f=L_1\times L_2$ that fits our data, then there is one of the form $f=L_1$. Accordingly our first few pruning rules are: remove a tree T from the list $\mathscr{T}$ of all gentrees with depth up to d if T has the subexpression

$$\text{[R1]} \quad L_1L_2 \text{ or } L_1/L_2. \tag{6}$$

$$\text{[R2a]} \quad L_1(L_3\pm L_4). \tag{7}$$

$$\text{[R2b]} \quad (L_1\pm L_2)(L_3\pm L_4). \tag{8}$$

$$\text{[R3]} \quad (L_1\pm L_2)/L_3. \tag{9}$$

The first rule was explained above. The second follows by associativity: $L_1(L_3+L4)=L_1L_3+L1L_4=L_1'+L_2'$, for some L-monomials L_1' and L_2'. If a T with a subexpression $L_1(L_3+L4)$ appears in $\mathscr{T}$, then replacing this subexpression by $L_1'+L_2'$ results in another gentree T' of the same depth, which must therefore be in $\mathscr{T}'$. The same idea can be applied to the subexpression $L_1(L_3-L_4)$. We can apply associativity twice to justify rule R2b, as $(L_1+L_2)(L_3+L_4)=L_1'+L_2'+L_3'+L_4'$, for some L_i' (the same argument holds when either $+$ is replied by a $-$ in

R2b). Once again, the second expression has the same depth as the first, and therefore there must be a tree in $\mathscr{T}$ containing it. R3 can be explained similarly.

Supplementary Note 2.3 - MINLP formulation for a gentree

Let the data points be $\mathbf{X}^{(i)}$ for $i \in I$, where I is an index set, and let the features/independent variables be $x_1, \ldots, x_n$. Let $\mathbf{Y}$ stand for the observations of the dependent variable y, with $Y^{(i)}$ standing for the i-th observation (of the dependent variable). Let T be a given gentree with m leaf nodes, and let $\mathbf{p}$ be the vector of all integer variables corresponding to the powers of the independent variables in the different leaf nodes. Then $\mathbf{p} \in \mathbb{Z}^{mn}$. Let $h_1, \ldots h_m$ be the variables corresponding to the constants in leaf nodes $1, \ldots, m$. Finally, assume we have a 0-1 variable z_i that determines whether or not the ith leaf node has a constant h_i that is different from one. Thus the (vector) variables in our model are $\mathbf{p}, \mathbf{z}$ and $\mathbf{h}$. Let $f_{\mathbf{h},\mathbf{p},\mathbf{z},T}$ stand for the symbolic expression defined by fixing the values of these variables. Then the MINLP we solve can be framed as:

$$\min \quad \sum_{i\in I}(Y^{(i)} - f_{\mathbf{h},\mathbf{p},\mathbf{z},T}(\mathbf{X}^{(i)}))^2 \tag{10}$$

$$\text{s.t.} \quad -\delta \le p_i \le \delta \quad \text{for } i = 1, \ldots, mn \tag{11}$$

$$-\Omega z_i + (1 - z_i) \le h_i \le \Omega z_i + (1 - z_i) \quad \text{for } i = 1, \ldots, m \tag{12}$$

$$\sum_{i=1}^{m} z_i \le k \tag{13}$$

$$\mathbf{z} \in \{0,1\}^m, \quad \mathbf{p} \in \mathbb{Z}^{mn} \tag{14}$$

The constraints (14) and (11) force z_i to take on 0-1 values, and p_i to take on values in the range $\{-\delta, -\delta + 1, \ldots, \delta\}$. The constraint (12) restricts h_i to lie in the range $[-\Omega, \Omega]$ if z_i has value 1, and forces h_i to take on value 1, when z_i has value 0. The constraints (13) allow at most k of the z_i variables to have value 1, and therefore at most k of the h_i values to be different from 1. The function $f_{\mathbf{h},\mathbf{p},\mathbf{z},T}$ is composed of the operators in the non-leaf nodes of T from the L-monomials in the leaf nodes. The objective function is the sum of squares of differences between the symbolic expression values for each input data point and the corresponding dependent variable value (call it the *least-square error*).

We use BARON to solve the MINLPs we generate, and thus T and $f_{\mathbf{h},\mathbf{p},\mathbf{z},T}$ are limited by the operators that BARON can handle $(+, -, \times, /, \exp(), \log())$. We will illustrate f for a few examples, rather than specify it formally. Suppose we are trying to derive the formula $F = G\frac{m_1 m_2}{r^2}$, where m_1, m_2 and r are independent variables, and G is an unknown constant, and we have multiple data points (for $i \in I$) with values for the independent variables, and for associated F. If T is a depth 0 gentree, then T is just a single L-monomial, and $f_{\mathbf{h},\mathbf{p},\mathbf{z},T} = hm_1^a m_2^b r_i^c$ where a, b, c are undetermined integers in the range $[-\delta, \delta]$, and h is an undetermined real number in the range $[-\Omega, \Omega]$. The objective function is then

$$\sum_{i\in I}(F_i - hm_{1i}^a m_{2i}^b r_i^c)^2 \ , \tag{15}$$

where F_i, m_{1i}, m_{2i} and r_i are the values of F, m_1, m_2, r in the ith data point. If the gentree can be written as $L_1 + L_2$, then the objective function becomes

$$\sum_{i\in I}(F_i - (h_1 m_{1i}^{a_1} m_{2i}^{b_1} r_i^{c_1} + h_2 m_{1i}^{a_2} m_{2i}^{b_2} r_i^{c_2}))^2 \ ; \tag{16}$$

here a_i, b_i, c_i and h_i are undetermined, and we solve for these values.

It is important to note that, as depicted in Supplementary Figure 2, an expression tree of a certain depth can sometimes be represented by a gentree of smaller depth. Therefore, by enumerating gentrees of a certain depth, one obtains a much richer class of functions than can be obtained by expression trees of the same depth.

Supplementary Note 2.4 - Enumeration and parallel processing

By enumerating the generalized expression trees and solving them separately, the problem is divided into multiple, easier-to-solve sub-problems (operator placement in non-leaf nodes does not have to be determined any more) that can be solved much more quickly. This "divide and conquer" formulation can obtain solutions to problems that were intractable for the formulation in the work of Austel et al. [14].

When available, we exploit parallelism, and run multiple threads, with one MINLP corresponding to a single gentree in a single thread. If we obtain a solution that fits the data within a prescribed tolerance from a gentree with depth d', then we stop all processes/threads containing gentrees with depth $> d'$. We terminate a gentree if the lower bound on the least-square error exceeds the least-square error found from other gentrees of the same or lower depth. Thus we execute a branch-and-bound type search, and implicitly search for the least depth gentree that fits the data.

If we have more gentrees than available cores, we process them in a round robin fashion; if t is the number of cores, we start solving for t gentrees in parallel, and after a fixed amount of time (10 seconds), we pause the first t gentrees, and start solving t more, till we either find a solution or run out of gentrees in which we case we start from the first gentree. The gentrees are sorted by a measure of complexity (roughly equal to the number of nodes).

Supplementary Note 2.5 - Scaling

The gentree enumeration running time (and thus that of our overall algorithm) grows exponentially with gentree depth d'. As described earlier, we enumerate a number of gentrees, and solve an MINLP per gentree. The number of distinct gentrees grows exponentially with the depth d' of the gentree and also grows rapidly with the number of operators used; see Supplementary Table 6.

Max tree depth	Number of trees with operators: $+, -, \times, /$	Number of trees with operators: $+, -, \times, /, \sqrt{}$
1	2	2
2	6	7
3	31	60
4	1153	4485
5	1,506,165	$\geq$ 10,000,000

Supplementary Table 6. Number of gentrees as a function of depth

The number of gentrees for $d' = 2,3,4,5$ are, respectively, 7, 60, 4485 and over ten million when we use the operators $+, -, \times, /, \sqrt{}$. Our gentree enumeration approach is unlikely to be tractable for $d' \geq 6$ and is already hard for $d' = 4$ if we include more operators than listed earlier. However, each L-monomial can represent large expression trees: for example, the L-monomial $hm_1^a m_2^b r_i^c$, which corresponds to a depth 0 gentree, needs a depth 3 binary expression tree if the powers a,b,c are integers between -2 and 2. Thus, our depth-3 L-monomial trees can easily need expression trees with depth 6 or more to represent them when we have three independent variables. If the number of independent variables in the L-monomial is more than 3, or the magnitude of the powers is increased, then even deeper expression trees would be needed.

Solving the MINLP problem associated with each gentree can itself be a hard problem. MINLP solvers such as BARON typically use a branch-and-bound algorithm to solve such a problem to a prescribed tolerance. Upon termination (assuming a minimization problem) BARON returns both a solution and a lower bound on all possible solution objective values such that the returned solution objective value and the lower bound differ by less than the prescribed tolerance. However, the number of branch-and-bound nodes – and the computing time – can grow exponentially. To avoid exponential growth, we terminate the process within a prescribed time limit and end up with a large gap between the objective value and the lower bound. In Supplementary Figures 3 and 4, we show how the upper bound (on the absolute error between the derived model and data) and lower bound change with time during the MINLP solution process along with the growth in number of branch-and-bound nodes. In Supplementary Figure 3a we observe that shortly after 500 seconds, BARON certifies that the optimal error is roughly 8.5. In Supplementary

Figure 4a we observe that the error drops to roughly 0.002 shortly before 300 seconds, but the process does not terminate as BARON is unable to improve the lower bound from 0 (and termination conditions are either the absolute gap between bounds should be 10^{-4} or the relative gap between bounds should be 10^{-3}). Consider the single MINLP associated with the gentree in Supplementary Figure 4, and assume we have 3 independent variables and the powers of each variable are constrained to be integers between 2 and -2 (see Supplementary Note 2.7). The number of possible discrete choices to be made (i.e., the power of each variable in each L-monomial) is at least $5^{15} \approx 30$ billion. BARON is able to obtain a solution of reasonable quality by examining about 120,000 subproblems.

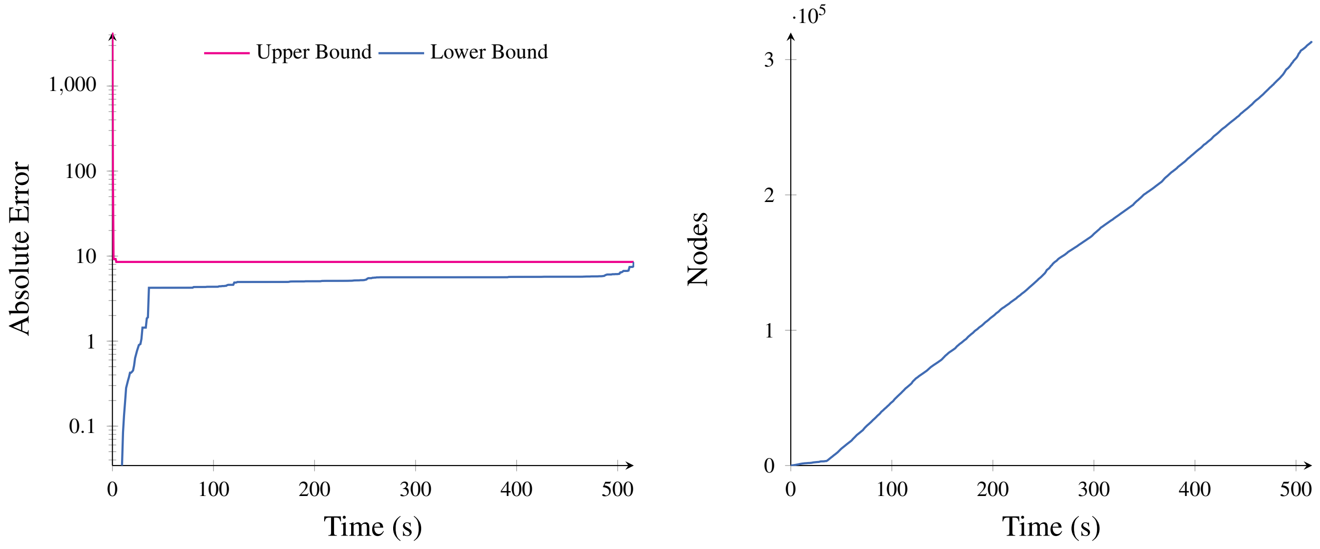

(a) Change with time in upper bound and lower bound values. **(b)** Growth in number of branch-and-bound nodes with time.

Supplementary Figure 3. MINLP properties over time, gentree for $L_1 + L_2 + L_3$.

Supplementary Note 2.6 - Dimensional analysis

Consider Newton's law of universal gravitation which says that the gravitational force between two bodies is proportional to the product of their masses and inversely proportional to the square of the distance between their centers (of gravity):

$$F \propto \frac{m_1 m_2}{r^2}$$

and the constant of proportionality is G, the gravitational constant. Therefore, we have

$$F = G\frac{m_1 m_2}{r^2}.$$

The units of G are chosen so that units of the right-hand-side expression equal the units of force (mass $\times$ distance / time-squared). Suppose one is given a data set for this example, where each data item has the masses of two bodies and the distance and gravitational force between them. Dimensional analysis would rule out $F = m_1 m_2/r^2$ or $F = m_1/m_2 + m_2 r$, for example, as possible solutions.

If constants can have units and every L-monomial can have a such a constant, then dimensional analysis conveys essentially no information. For example, we can choose constants h_1, h_2, h_3 with appropriate dimensions so that both the expressions $F = h_1 m_1 m_2/r^2$ and $F = h_2 m_1/m_2 + h_3 m_2 r$ satisfy all dimensional requirements. We allow constants to have units or not based on an input flag.

We next explain the constraints we add to our formulation to enforce dimensional consistency. Assume that all constants have no units, and that $k = 2$, and $\delta = 2$, and $d = 1$. For the gravitation example (without the gravitational constant G as an input), each L-monomial L has the form

$$L = h m_1^a m_1^b r^c, \tag{17}$$

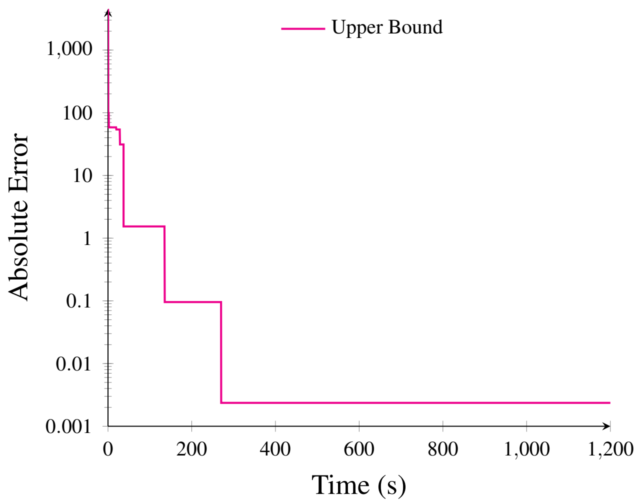

(a) Change with time in upper bound (the lower bound remains zero throughout).

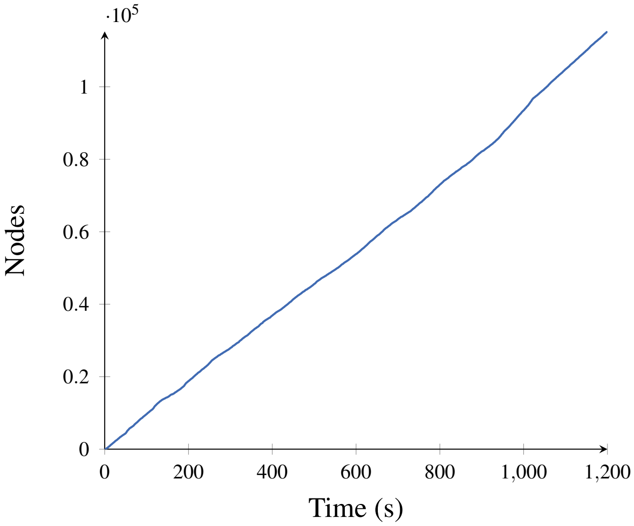

(b) Growth in number of branch-and-bound nodes with time.

Supplementary Figure 4. MINLP properties over time, gentree for $\left(\sqrt{(L_1+L_2)}\,(L_3+L_4)\right)/L_5$.

where a, b and c are bounded integer variables with an input range of $[-2,2]$, and h is a variable representing a constant. We add to the system of constraints (11) - (14) linear constraints that equate the units of the symbolic expression to those of the dependent variable. For the case our gentree consists of a single node (as in the first tree in Supplementary Figure 2), our symbolic expression has the form (17), and we add the linear constraints

$$a\begin{bmatrix}1\\0\\0\end{bmatrix}+b\begin{bmatrix}1\\0\\0\end{bmatrix}+c\begin{bmatrix}0\\1\\0\end{bmatrix}=\begin{bmatrix}1\\1\\-2\end{bmatrix}.$$

Here, each component of a column vector corresponds to a unit (mass, distance, time, respectively). The column on the right-hand side represents the units of force, i.e., mass times distance divided by squared time. The columns on the left-hand side represent the dimensionalities of mass and distance, respectively. The third of the above linear equations does not have a solution.

Next, assume the symbolic expression is L_1+L_2 where $L_1=h_1 m_1^{a_1} m_2^{b_1} r^{c_1}$ and $L_2=m_1^{a_2} m_2^{b_2} r^{c_2}$, and the unkowns are a_i, b_i, c_i and h_i. The units of L_1 and L_2 have to match, and must also equal the units of F. The linear constraints we add are

$$a_1\begin{bmatrix}1\\0\\0\end{bmatrix}+b_1\begin{bmatrix}1\\0\\0\end{bmatrix}+c_1\begin{bmatrix}0\\1\\0\end{bmatrix}=\begin{bmatrix}1\\1\\-2\end{bmatrix} \tag{18}$$

$$a_2\begin{bmatrix}1\\0\\0\end{bmatrix}+b_2\begin{bmatrix}1\\0\\0\end{bmatrix}+c_2\begin{bmatrix}0\\1\\0\end{bmatrix}=\begin{bmatrix}1\\1\\-2\end{bmatrix}. \tag{19}$$

Finally, for the symbolic expression $L_1 \times L_2$, to compute the units of this expression we need to add up the units of L_1 and of L_2. Thus we sum the left hand sides of (18) and (19) and equate this sum to the right hand side of (18). We can similarly deal with dimension matching in the remaining gentrees of depth 1 via linear constraints. For greater depth gentrees, we apply the ideas above to depth 1 (non-leaf nodes), and then to depth 2 nodes and so on.

Supplementary Note 2.7 - Settings for experiments

Because of the exponential worst-case scaling behavior of our method, we choose specific parameters that allow our method to terminate in a reasonable amount of time.

We set the depth limit d to 3. We experiment with different values of k, the number of constants, for each dataset. We terminate when we find an expression with objective value (squared error) less than 10^{-4} (error tolerance). The operators we use are described in Supplementary Note 2.1. We set Ω to 100, so all constants are in the range $[-100, 100]$. We set $\delta = 2$ for computational efficiency. This means that each power in an L-monomial lies in the range $[-2, 2]$. Under this setting, the pruning rules – R1, R2,R3– remove potentially non-redundant gentrees, and our algorithm does not explore all possible symbolic expressions that are representable as a gentree of depth up to 3. Though L-monomials are closed under multiplication, L-monomials with bounded powers are not. For example, if there is a solution of the form $x_1^3 x_2^2$, we may not find it.

We constrain the search in three more ways. We bound the sum of the variable powers in an L-monomial by an input number $\tau = 6$. Thus, for each term of the type (5), we add the constraint $\sum_{i=1}^{n} |a_i| \leq \tau$, which is representable by a linear constraint using n auxiliary variables $a_i' : \sum_{i=1}^{n} a_i' \leq \tau$ and $a_i' \geq 0, -a_i \leq a_i'$ and $a_i \leq a_i'$ for $i = 1, \ldots, n$. Secondly, we add another pruning rule. We remove any gentree which contains a subexpression of the form $\sqrt{L}$ where L is an L-monomial (we allow $\sqrt{L_1 + L_2}$, for example). We use dimensional analysis when feasible, other than when we perform a comparison with other SR codes (for example in Supplementary Tables 13 - 15) which do not use dimensional analysis.

Supplementary Note 3 - Reasoning and Derivability

Supplementary Note 3.1 - Logic and Reasoning Background

We assume the reader has knowledge of basic first-order logic (FOL) and automated theorem proving terminology and thus will only briefly describe the terms commonly seen throughout this paper (for readers interested in learning more about logical formalisms and techniques see [19, 20]).

In this work, we focus on *First-Order Logic (FOL) formulae* with equality and basic arithmetic operators, and so are defined based on the FOL grammar with the addition of the function symbols for equality $=$, inequality $>$, $<$, sum $+$, subtraction $-$, product $\times$, division $/$, power ^ (the square root is interpreted as a power of $\frac{1}{2}$) and absolute value $|\cdot|$.

First-order logic formulae are formal expressions based on an alphabet of predicates, functions, and variable symbols which are combined by logical connectives. A term is either a variable, a constant (a function with no arguments), or, inductively, a function applied to a tuple of terms. A formula is either a predicate applied to a tuple of terms or, inductively, a connective (e.g., $\wedge$ read as "and", $\vee$ as "or", $\neg$ as negation, etc.) applied to some number of formulae. In addition, variables in formulae can be universally or existentially quantified (i.e., by the quantifiers $\forall$ and $\exists$ which read as "for all" and "exists" respectively), where a quantifier introduces a semantic restriction for the interpretation of the variables it quantifies.

In the standard FOL problem-solving setting, an Automated Theorem Prover (ATP), also called a reasoner, is given a *conjecture*, that is, a formula to be proved true or false, and *axioms*, that is, formulae known to be true. The set of axioms is also called the *background theory*. By application of an inference rule, a new true formula can be derived from the axioms. This operation can be repeated, including the use of prior derived formulae; this yields a sequence of new true formulae, called a *derivation*. This is done until the given conjecture appears among the derived formulae; the sequence of applied rules and formulae along the way comprise a *proof* of the given conjecture. The set of all *derivable formulae* from a background theory is the set of all logic formulae that can be derived from the set of axioms defining the background theory.

Supplementary Note 3.2 - KeYmaeraX

Reasoning software tools for arithmetic and calculus that, in principle, have the required logic capabilities of checking consistency, validation, and deduction include for example SymPy [21], Prolog [22], Mathematica [23], Beagle [24], and KeYmaera [25, 26].

We integrated in our system several reasoning tools for arithmetic and calculus, analyzing their expressiveness and capabilities (including, but not only, the one mentioned above). Our findings made us decide in favour of the KeYmaeraX reasoner, an automated theorem prover for hybrid systems that combines different types of reasoning: deductive, real algebraic, and computer algebraic reasoning. The underlying logic supported by KeYmaera is differential dynamic logic [27], which is a real-valued first-order dynamic logic for hybrid programs. KeYmaera is based on a generalized free-variable sequent calculus inference rule for deductive reasoning and has an underlying CAD system (e.g. Mathematica).

KeYmaeraX provides fast computation for first-order logical formulae combined with arithmetic and differential equations, even though it does not provide full explainability for the derivations, meaning that only the final state (provable/not-provable) is available, while the proof-steps are not.

Supplementary Note 3.3 - KeYmaeraX formulation for Kepler's third law

We first consider the KeYmaera formulation† for the β_{∞}^{r} error. As an example, we consider $f(\mathbf{x})$ corresponding to $p = \sqrt{0.1319 d^3}$ extracted via SR from the solar system dataset. We compute the error between this function and $f_{\mathscr{B}}$, represented by p, which is the variable corresponding to the orbital period‡.

```
Problem (( m1>0 & m2>0 & p>0 & d2>0 & d1>0 & m1N>0 & m2N>0 & pN>0
    & G = (6.674 * 10^(-11)) & pi = (3.14) & err = 10^-2
    & ( m1 * d1 = m2 * d2 )
    & ( d = d1 + d2 )
    & ( Fg = (G * m1 * m2) / d^2 )
    & ( Fc = m2 * d2 * w^2 )
    & ( Fg = Fc )
    & ( p = (2 * pi )/w )
    & convP = (1000 * 24 * 60 * 60 )
    & convm1 = (1.9885 * 10^30 )
    & convm2 = (5.972 * 10^24)
    & convD = (1.496 * 10^11)
    & m1N = m1 / convm1
    & m2N = m2 / convm2
    & dN = d / convD
    & pN = p / convP
   )->(((m1N=1 & m2N=0.055 & dN=0.3871) ->
              abs((0.1319 * dN^3 )^(1/2) - (pN))/pN < err)
     & ((m1N=1 & m2N=0.815 & dN=0.7233) ->
              abs((0.1319 * dN^3 )^(1/2) - (pN))/pN < err)
     & ((m1N=1 & m2N=1 & dN=1) ->
              abs((0.1319 * dN^3 )^(1/2) - (pN))/pN < err)
     & ((m1N=1 & m2N=0.107 & dN=1.5237) ->
              abs((0.1319 * dN^3 )^(1/2) - (pN))/pN < err)
     & ((m1N=1 & m2N=317.8 & dN=5.2044) ->
              abs((0.1319 * dN^3 )^(1/2) - (pN))/pN < err)
     & ((m1N=1 & m2N=95.159 & dN=9.5826) ->
              abs((0.1319 * dN^3 )^(1/2) - (pN))/pN < err)
     & ((m1N=1 & m2N=14.536 & dN=19.2184) ->
              abs((0.1319 * dN^3 )^(1/2) - (pN))/pN < err)
     & ((m1N=1 & m2N=17.147 & dN=30.07) ->
              abs((0.1319 * dN^3 )^(1/2) - (pN))/pN < err)
) ) End.
```

†We omit Variables and Definitions from the KeYmaeraX formulation for simplicity.

‡Note that in the KeYmaeraX formulation we add the suffix N to the variables after they have been normalized (e.g., p become pN, m_1 become m1N, d become dN, etc.). In the text description we use the original variable names for simplicity.

In particular we see that:

- **line 1** describes the feasibility constraints, e.g., the masses only admit positive values;
- **line 2** defines some constants such as the gravitational constant, the value of π (`pi`), and the error bound (`err` $= 10^{-2}$);
- **lines 3-8** are the axioms of the background theory;
- **lines 9-16** are the normalization specifications (e.g. `m1N` is the normalized variable corresponding to the mass $\mathtt{m}_1$);
- **lines 17-24** contain the specification of the β^r_∞ error: given each data point (which specifies the value of the two masses and their relative distance) we want to prove that the distance between the formula induced from the data (in this case $p = \sqrt{0.1319d^3}$) and the derivable formula for `p` is smaller than `err`.

KeYmaera is able to produce a successful proof of the formulation above for an error bound of `err` $= 10^{-2}$.

The value for the reasoning error β^r_∞ is computed by using binary search over the values of the relative error $|(\sqrt{0.1319d^3} - p)|/p$. We used a time limit of 1200 seconds (20 min) and stopped the binary search process when a precision of 10^{-4} is attained.

The formulation for β^r_2 error is very similar, with the difference that the binary search is performed over the single data points as described in Algorithm 1, where $\min_e \{\text{KeYmaera}\ell_2(i,e) \mid \mathtt{precision}\}$ computes an upperbound on the minimum value for e for a give precision level (which is 10^{-4} in the experiments) such that KeYmaera$\ell_2(i,e)$ returns $true$. This is necessary because KeYmaera is fundamentally a boolean function, mapping formulations to a binary (success/failure) state. The minimum is therefore approximated, via binary search in a given interval, for given precision level and time limit.

Algorithm 1 β^r_2 computed with KeYmaera

```
procedure β₂ʳ(Dataset)
    for i in Dataset do
        e_i ← min_e {KeYmaeraℓ₂(i,e) | precision}
    β₂ʳ ← sqrt(e_i² + ··· + e_n²)
return β₂ʳ
```

procedure β^r_2(`Dataset`)
  for i in `Dataset` **do**
    $e_i \leftarrow \min_e \{\text{KeYmaera}\ell_2(i,e) \mid \mathtt{precision}\}$
  $\beta^r_2 \leftarrow \sqrt{e_i^2 + \cdots + e_n^2}$
return β^r_2

The KeYmaera formulation for KeYmaera$\ell_2(i,e)$, on a specific data point i and an error bound e, is a Boolean function that returns $true$ if the following program is provable and $false$ otherwise. KeYmaera$\ell_2(i,e)$ returns $true$ when, for a given set of axioms, the absolute value of the relative distance between a given function and a derivable one is smaller than e for a given data point i. For example, given the data point $(m_1, m_2, d) = (1, 0.055, 0.3871)$ and error $e = 10^{-2}$ we have the following formulation:

```
1  Problem (( m1>0 & m2>0 & p>0 & d2>0 & d1>0 & m1N>0 & m2N>0 & pN>0
2      & G = (6.674 * 10^(-11)) & pi = (3.14) & e = 10^-2
3      & ( m1 * d1 = m2 * d2 )
4      & ( d = d1 + d2 )
5      & ( Fg = (G * m1 * m2) / d^2 )
6      & ( Fc = m2 * d2 * w^2 )
7      & ( Fg = Fc )
8      & ( p = (2 * pi )/w )
9      & convP = (1000 * 24 * 60 * 60 )
10     & convm1 = (1.9885 * 10^30 )
11     & convm2 = (5.972 * 10^24)
12     & convD = (1.496 * 10^11)
```

```
    & m1N = m1 / convm1
    & m2N = m2 / convm2
    & dN = d / convD
    & pN = p / convP
   )->(((m1N=1 & m2N=0.055 & dN=0.3871) ->
              abs((0.1319 * dN^3 )^(1/2) - (pN))/pN < e)
) ) End.
```

We observe that the point-wise reasoning errors are not very informative if SR yields a low-error candidate expression (measured with respect to the data), and the data itself satisfies the background theory up to a small error, which indeed is the case with the data we use; the reasoning errors and numerical errors are very similar. This is true because, if we can evaluate the numerical error, then we may assume that the error of the candidate formula at the data points is substantially equal to the error of the correct formula. In particular, this is true when the data is generated synthetically like in the Feynman Symbolic Regression Database [28].

Let's consider again the formula $\sqrt{0.1319d^3}$ (extracted via SR from the solar system dataset) for the variable of interest p. The KeYmaera formulation for the generalization relative reasoning error $\beta^r_{\infty,S}$ is the following:

```
1  Problem (( p>0 & pN>0
2     & G = (6.674 * 10^(-11)) & pi = (3.14) & err = 10^-6
3     & m1N>=0.08 & m1N<=1
4     & m2N>=0.0002 & m2N<=1
5     & dN>=0.0111 & dN<=30.1104
6     & ( m1 * d1 = m2 * d2 )
7     & ( d = d1 + d2 )
8     & ( Fg = (G * m1 * m2) / d^2 )
9     & ( Fc = m2 * d2 * w^2 )
10    & ( Fg = Fc )
11    & ( p = (2 * pi )/w )
12    & convP = (1000 * 24 * 60 * 60 )
13    & convm1 = (1.9885 * 10^30 )
14    & convm2 = (5.972 * 10^24)
15    & convD = (1.496 * 10^11)
16    & m1N = m1 / convm1
17    & m2N = m2 / convm2
18    & dN = d / convD
19    & pN = p / convP
20   )->((abs(0.1319 * dN^3 )^(1/2) - pN ) / (pN) < err ))
21 End.
```

In particular we have that:

- **line 1** describes the feasibility constraints;
- **line 2** defines some constants such as the gravitational constant, π value and the error bound;
- **lines 3-5** define the intervals S for the variables, extracted from the normalized data points in the dataset (e.g. the planets in the solar system have a distance from the sun that is between 0.0111 and 30.1104 astronomical units);
- **lines 6-11** are the axioms of the background theory;
- **lines 12-19** are the normalization specifications;
- **lines 17-24** contain the specification of the error $\beta^r_{\infty,S}$: given each data point (instantiation of the value of the two masses and their relative distance) we want to prove that the distance between the formula induced from the data (in this case $\sqrt{0.1319d^3}$) and the derivable formula for p is smaller than `err`.

Supplementary Note 3.4 - KeYmaera formulation for relativistic time dilation

We give a formulation to check for the quality of generalization for a given formula, in particular, the function $y = -0.00563v^2$ computed from data. The velocity of light c is given as a fixed constant $3 \cdot 10^8$ (meters per second). The generalized absolute reasoning error $\beta^a_{\infty,S}$ is bounded above by 1. The following formulation checks if all the derivable formulae differs (after scaling by 10^{15}) from the function $-0.00563v^2$ by at most 1 in the domain S defined by $37 \leq v \leq 115$.

```
Problem((d > 0 & L > 0 & c = 3*10^8 & err = 1
      & v >= 37 & v <= 115
      & dt0 = 2*d/c
      & L^2 = d^2 + (v*dt/2)^2
      & dt = 2*L/c
      & f0 = 1/dt0
      & f = 1/dt
      & df = f - f0
      & y = 10^15*df/f0 )
      -> ( ( abs( (-0.00563)*(v^2)) - y < err ) ) )
End.
```

Supplementary Note 3.5 - KeYmaeraX formulation for proving Langmuir's adsorption equation

The KeYmaeraX formulation to prove the Langmuir equation:

$$q = \frac{S_0(k_{\text{ads}}/k_{\text{des}})p}{1+(k_{\text{ads}}/k_{\text{des}})p}. \tag{20}$$

from the background theory described in the manuscript (L1–L5) is the following:

```
Definitions
 Real kads;
 Real kdes;
 Real S0;
End.
Problem
( \forall Q \forall S \forall Sa \forall P \forall rdes \forall rads(
      (kads>0 & kdes>0 & S0>0 & Q>0 & S>0 & Sa>0 & P>0 & rdes>0 & rads>0
      & S0 = S + Sa
      & rads = kads * P * S
      & rdes = kdes * Sa
      & rads = rdes
      & Q = Sa)
      -> Q = ((S0 * (kads / kdes)) * P) / (1 + ((kads / kdes)*P) ) ))
End.
```

This formulation is successfully proved by KeYmaera.

As expected, when removing one or more axioms that are strictly necessary to prove the theorem (e.g., the axiom $r_{\text{des}} = k_{\text{des}}S_{\text{a}}$), it is not possible to prove the formula anymore. Similarly, if f does not correspond to the correct formula (while the axiom set is complete and correct) KeYmaera is not able to provide a provability certification. In this case it is possible to create a numerical counterexample (a numerical assignment to all the variables involved in the problem formalization, that falsify the implication). Moreover, with the addition of redundant or unnecessary axioms we are still able to prove the conjecture.

An example of a counterexample, obtained by removing an essential axiom from the background theory (the axiom $r_{\text{des}} = k_{\text{des}}S_{\text{a}}$), is the following:

$$[Q,S,P,S_0,r_{des},S_a,k_{des},k_{ads}r_{ads}] = [1,1,1/2,2,1/2,1,1,1,1/2]$$

It is possible to add additional constraints and assumptions to generate further counterexamples: e.g., if we exclude the value 1 for the variable Q (the assignment of Q in the previous counterexample) adding the constraint $Q \mathrel{!}= 1$, the Counter-Example Search tool provides a new solution:

$$[Q, S, P, S_0, r_{des}, S_a, k_{des}, k_{ads} r_{ads}] = [1/4, 1/4, 1/2, 1/2, 1/8, 1/4, 1, 1, 1/8]$$

Langmuir defined expressions to model adsorption onto a material that contains different kinds of sites, with different interaction strengths [5]. Consequently, we generalize the background theory to model this scenario. Some quantities (S_{a}, S_0, S, k_{ads}, k_{des}, r_{ads}, and r_{des}) now depend on the site. The axioms below, which are universally quantified over the sites, hold for each site as before, while the definition of the adsorbed amount changes to allow for the presence of multiple sites N. The new set of axioms is:

GL1. $\forall X.\, S_0(X) = S(X) + S_a(X)$

GL2. $\forall X.\, r_{ads}(X) = k_{ads}(X) P S(X)$

GL3. $\forall X.\, r_{des}(X) = k_{des}(X) S_a(X)$

GL4. $\forall X.\, r_{ads}(X) = r_{des}(X)$

GL5. $Q = \sum_{i=1}^{N} S_a(X)$

GL6. $\bigwedge_{i=1}^{N} \left(St = i \rightarrow (\bigwedge_{j>i} S_0(j) = 0)\right)$

GL7. $\bigvee_{i=1}^{N} St = i$

where GL1–GL4 are as before; GL5 ensures that the total amount adsorbed is the sum of the amounts adsorbed in each site; GL6 ensures that if there are i site types ($St = i$) the types with higher index have no sites; and GL7 ensures that there is only one value for the number of site types.

We implemented an alternative KeYmaera formulation for the two-sites model:

```
Definitions
  Real kads_1;
  Real kdes_1;
  Real S0_1;
  Real kads_2;
  Real kdes_2;
  Real S0_2;
End.
ProgramVariables
  Real Q;
  Real S_1;
  Real S_2;
  Real Sa_1;
  Real Sa_2;
  Real P;
  Real rdes_1;
  Real rads_1;
  Real rdes_2;
  Real rads_2;
End.
Problem
( ( kads_1>0 & kdes_1>0 & S0_1>0 & S_1>0 & Sa_1>0 & rads_1>0 & rdes_1>0
      & kads_2>0 & kdes_2>0 & S0_2>0 & S_2>0 & Sa_2>0 & rads_2>0 & rdes_2>0 &
      & Q>=0 & P>0
      & S0_1 = S_1 + Sa_1
      & rads_1 = kads_1 * P * S_1
      & rdes_1 = kdes_1 * Sa_1
```

```
     & rads_1 = rdes_1
     & S0_2 = S_2 + Sa_2
     & rads_2 = kads_2 * P * S_2
     & rdes_2 = kdes_2 * Sa_2
     & rads_2 = rdes_2
     & Q = Sa_1 + Sa_2 )
   -> Q = (S0_1 * (kads_1 / kdes_1) * P)/(1+(kads_1/kdes_1) *P)
   +(S0_2 * (kads_2/ kdes_2) * P)/ (1 + ((kads_2 / kdes_2) * P)))
End.
```

This formulation is successfully proved by KeYmaera and is an instance of the formulation described for a generic number of sites. This is done by a duplication of variables used in the axiom system for the one-site model. This grounding of the general formulation (for N sites) helps the reasoner converge faster to a solution if the number of sites is small. However, this approach applied to n sites would result in an exponential growth (with n) of the number of formulae and would lead to increasing computing times. In general, a more compact representation (with fewer logical/non-logical symbols) is preferred.

When the input formula (to be derived) has numerical constants, we need to introduce existentially quantified variables. For example, given an input formula $P/(0.00927\,P+0.0759)$, we first transform it to $P/(c_1\,P+c_2)$ (where c_1 and c_2 are existentially quantified). The following KeYmaera formulation is used:

```
Definitions
  Real kads;
  Real kdes;
  Real S0;
End.
Problem
( \exists c1 \exists c2 \forall Q \forall S
   \forall Sa \forall P \forall rdes \forall rads(
    (kads>0 & kdes>0 & S0>0 & Q>0 & S>0 & Sa>0 & P>0 & rdes>0 & rads>0
    & S0 = S + Sa
    & rads = kads * P * S
    & rdes = kdes * Sa
    & rads = rdes
    & Q = Sa)
    -> ( c1>0 & c2>0 & Q = P/(c1 * P +c2) )))
End.
```

This formulation is successfully proved by KeYmaera , however it does not provide any (symbolic or numerical) instantiation for the existentially quantified variables that satisfy the logic program. In this case, manual inspection can show that instantiating $c_1 = 1/S_0$ and $c_2 = \frac{k_{\mathrm{des}}}{k_{\mathrm{ads}}S_0}$ provides a valid solution. To obtain this result automatically would require an extension of KeYmaeraX (or more broadly a theorem prover) to allow for explicit variable assignments.

Likewise, the formulation for proving expressions generated by symbolic regression with the two-site Langmuir model is the following:

```
Definitions
  Real kads_1;
  Real kdes_1;
  Real S0_1;
  Real kads_2;
  Real kdes_2;
  Real S0_2;
End.
```

```
Problem
( \exists c1 \exists c2 \exists c3 \exists c4 \forall Q
  \forall S_1 \forall S_2 \forall Sa_1 \forall Sa_2 \forall P(
    ( S_1>0 & Sa_1>0 & S0_1>0 & kads_1>0 & kdes_1>0
    &  S_2>=0 & Sa_2>=0 & S0_2>0 & kads_2>0 & kdes_2>0
    & Q>=0 & P>0
    & S0_1 = S_1 + Sa_1
    & kdes_1 * Sa_1 = kads_1 * P * S_1
    & S0_2 = S_2 + Sa_2
    & kdes_2 * Sa_2 = kads_2 * P * S_2
    & Q = Sa_1 + Sa_2)
    -> (Q = (c1*P^2+c2*P)/(P^2+c3*P+c4) & c1>0 & c2>0 & c3>0 & c4>0 )))
End.
```

Using this formulation, KeYmaera timed out without proving or disproving any of the conjectures provided by the SR module. However, we noticed that g_5 and g_7, of the form $q = \frac{c_1 p^2 + c_2 p}{p^2 + c_3 p + c_4}$, satisfy all the thermodynamic constraints $\mathscr{K}$ (in contrast to the other 4-parameter formulae), so we tried to prove them manually. We were able to do so, obtaining the following variable instantiations: $c_1 = S_{0,1} + S_{0,2}$, $c_2 = \frac{S_{0,1}K_1 + S_{0,2}K_2}{K_1 K_2}$, $c_3 = \frac{K_1 + K_2}{K_1 K_2}$, and $c_4 = 1/(K_1 K_2)$, where $K_i = k_{\text{ads},i}/k_{\text{des},i}$.

Supplementary Note 4 - Additional related work

Symbolic discovery of formulae is a well studied research field, and it is a recognised challenge for the entire Artificial Intelligence community [29].

We addressed the most relevant literature in the introduction to the paper. Some other works worth mentioning are at the intersection of symbolic discovery and deep learning. Several attempts have been made to use deep neural network for learning or discovering symbolic formulae [30–36]. For example, in the work by Iten et al. [35] the authors focused on modelling a neural network architecture on the human physical reasoning process. In the work by Arabshahi et al. [33] the authors use tree LSTMs to incorporate the structure of the symbolic expression trees, and combine symbolic reasoning and function evaluation for solving the tasks of formula verification and formula completion. Symbolic regression has been used [36] to extract explicit physical relations from components of the learned model of a Graph Neural Network (GNN). In a work by Derner et al. [37], the authors employ symbolic regression to construct parsimonious process models described by analytic formulae for real-time RL control when dealing with unknown or time-varying dynamics. In AI Feynman [28] (and the subsequent paper AI Feynman 2.0 [38]), the authors introduce a recursive multidimensional symbolic regression algorithm that combines neural network fitting with a suite of physics-inspired techniques to find an formula that matches data from an unknown function. In the work by Jin et al. [39], symbolic regression is combined with Bayesian models. Moreover, neuro-symbolic systems have received a lot of attention in the past years: see the papers on combining mathematical reasoning with deep neural networks [40, 41]. Bayesian program synthesis has also been successful for learning a library of formulae [42]. Other types of search have been investigated as well, e.g. Bayesian Markov chain Monte Carlo search [43], graph-based search [44], methods for identifying linear combinations of nonlinear descriptors for dynamic systems (SINDy) [45] or material property prediction (SISSO) [46]. However, to the best of our knowledge, these methods have never been combined with logical reasoning.

The use of logic constraints to help with the discovery of formulas from numerical data is a well known technique. The LGML tool of Scott et al. 2021 [47] is a pipeline involving a learning component (either a symbolic regression tool or a deep neural network) and a consistency checking component. The consistency checking is performed against a set of equities or inequalities describing the functional form that they are trying to learn: “logical equation in terms of the feature space and the unknown function”. The LGGA tool of Ashok et al. [48] is an extension of LGML and is a genetic algorithm enhanced by auxiliary truth in the form of “mathematical expressions that capture domain specific knowledge or simple properties of an unknown function”. The main goal of the consistency checking component of these two works is to understand if a set of constraints are satisfied by a given function

f' (the approximation of the correct function f). They thus check if $f' \models C$. Our work differentiate from these two methods as follows: 1) The type of constraints used is fundamentally different: we use general scientific laws describing the environment, often not including any of the variables present in the data, while in LGML and LGGA the authors only consider simple constraints on the functional form. 2) The reasoning task we are trying to solve is different: as mentioned above LGGA and LGML solve the logic task of $f' \models C$ where f' is an approximation of the function f and C is a single constraint on f functional form. AI-Descartes solve the task of $B \models f'$, where B is a collection of axioms and f' is an approximation of the function f. Moreover we introduce novel reasoning tasks such as the computation of the reasoning errors etc. A similar, however earlier, approach is the one by Bladek and Krawiec [49] where the authors formalize the task of symbolic regression with formal constraints by the generation of counterexamples. Another work that combines SR and prior knowledge is the work of Kubalik et al. [50, 51], where the authors consider a set of nonlinear inequality and equality constraints on the functional form of the function to discover. They add this prior knowledge in their multi-objective symbolic regression approach as a set of discrete additional data samples on which candidate models are exactly checked. In this way consistency checking can be incorporated into the fitness evaluation or as an additional optimization objective. Finally, the work of Engle and Sahinidis [52] introduce a novel deterministic mixed-integer nonlinear programming formulation for symbolic regression that uses derivative constraints through auxiliary expression trees.

Using logic constraints as part of ML tools increased in popularity in recent years in the Neuro-Symbolic field: e.g. using the violation of logic constraints as part of the loss of an NN [53, 54], or in other techniques [55–62]. However all these methods are based only on constraints describing the functional form that has to be learnt, and do not incorporate background-theory axioms (logic constraints that describe the other laws and variables that are involved in the phenomenon). Another Neuro-Symbolic research area that is relevant to our work is the extraction of logical rules from data (also called ILP or rule induction). Some examples are the work of Sen et al. [63] or Evans and Grefenstette [64] among many others [65, 66]. A related topic with a lot of attention is program synthesis. Recently there have been a lot of discussion on this topic in the context of Neuro-symbolic systems. Some example are the works of Nye et al. [67], Parisotto et al. [68], Valkov et al. [69] or Yang et al. [70].

Supplementary Note 5 - Comparison with other systems

We compared our system with some state-of-the-art methods on the three real-life problems (and associated datasets) that we considered in this work. In addition, we also performed a comparison on 81 problems out of 100 from the Feynman Symbolic Regression Database [28]; the associated formulas are taken from the Feynman lectures on physics, and we ignored the 19 that contain trigonometric functions. To create the data for each of the 81 problems, we chose 10 datapoints from each (synthetic) dataset and added 1% error (in the manner described in [28]), thereby creating small, noisy datasets to resemble the real-life data that we use. We note that the original dataset consists of 100,000 data points with zero error for each problem.

The list of systems we compared against is not meant to be comprehensive, but is a representative sample of state-of-the-art methods. We excluded some systems from the list because they were either not applicable (e.g., LGGA[§] [48]), not freely licensed (e.g., Eureqa [8]) or because the code was not available (e.g., LGML [47]).

The systems we considered are the following:

- **AI Feynman** [28, 38] is a symbolic regression algorithm that combines deep learning techniques with the exploitation of some characteristics typically present in functions studied in physics such as the presence of units, the use of low-order polynomials, symmetry, etc. The algorithm combines multiple modules that perform dimensional analysis, polynomial fit, brute-force enumeration, and neural network fit (and a few other tasks) to produce a list of candidate formulas.

[§] The comparison with LGGA was only possible for Langmuir's problem, since the system requires constraints on the functional form of f. We defined such constraints – the constraints $\mathscr{K}$ – only for Langmuir's problem. However, the only constraint that is supported by LGGA is $f(0) = 0$. The other constraints we use (e.g., monotonicity and limiting properties) are not currently supported by LGGA. We were thus unable to obtain good results with the LGGA tool and therefore decided not to report them.

- **TuringBot** [9] is a simulated annealing method to find expressions that fit the input data. The free version of the software allowed us to consider a maximum of 2 independent variables per problem. For Langmuir's adsorption equation and for the relativistic time dilation problem two independent variables are enough. For Kepler's third law, two independent variables are enough except for the Binary Stars dataset; therefore, we do not report results with this dataset.

- **PySR** [71, 72] is a tool that uses regularized evolution, simulated annealing, and gradient-free optimization to search for equations that fit the input data.

- **Bayesian Machine Scientist (BMS)** [43] is a Markov chain Monte Carlo based method that explores the space of possible models, based on prior expectations learned from a large empirical corpus of mathematical expressions.

The above systems return a score (in some cases this corresponds to the error) and the complexity for each output formula. We applied standard methods to select the best candidates, such as computing the Pareto front and identifying knee points on it, a common technique to choose from the Pareto front candidates [9, 73]. We used an existing python library, called *kneed* [74], that uses the kneedle algorithm [75] to compute the knee/elbow points of a function defined by a set of points. The knee point is the point of maximum curvature on such function. The systems PySR and Bayesian Machine Scientist return a best candidate and, therefore, for those methods we didn't identify the knee points.

The results for the different datasets are reported in Supplementary Tables 7 – 12. We show the Pareto front solutions for each method as well as the best candidate formula in the Pareto front (either the knee point or the best candidate formula returned by the corresponding system). Supplementary Figures 5 – 8 report the comprehensive set of results for TuringBot, AI Feynman, PySR and Bayesian Machine Scientist respectively, for two of the datasets (Kepler solar and Langmuir [5, Table IX]).

As we can see from the results, the systems very rarely produce the correct formula as the best candidate. However, some systems output the correct formula in the top candidates, but not all of them. Even in the case in which the correct formula appears in the top candidates, the systems do not have a method to identify the right formula in this list. The application of the reasoning component of our system would definitively help to identify the right one. The output of the systems is produced by using the default parameters configuration provided with the code (more details in Supplementary Table 16). A fine-tuning of these parameters for each dataset could lead to better results. However, a general case, in which the correct formula is unknown, would not allow the selection of the best parameters, so we think that using default parameters is more realistic.

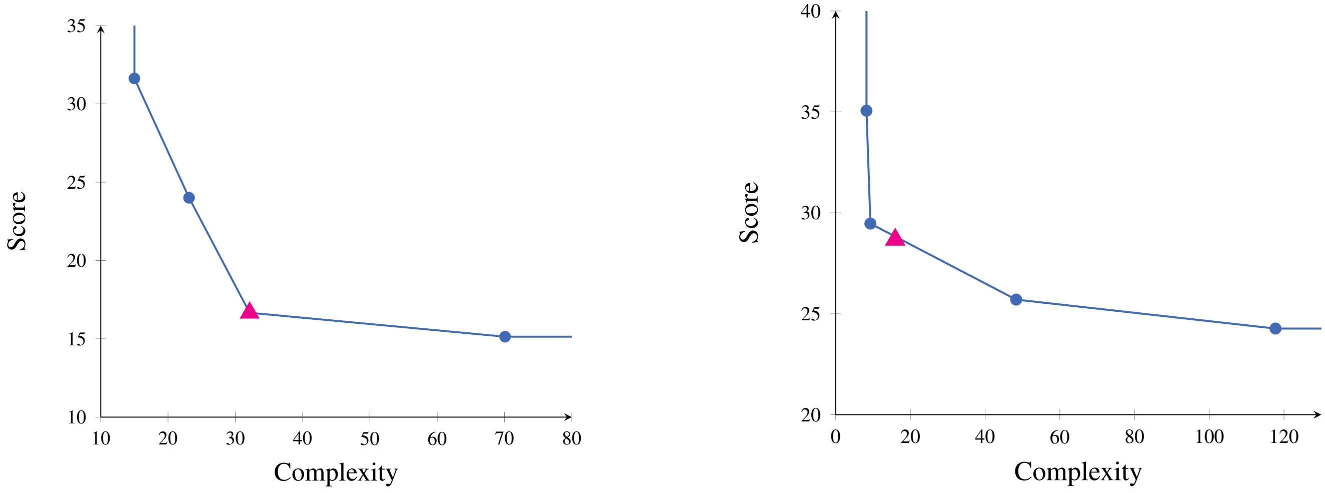

(a) Kepler Solar

(b) Langmuir [5, Table IX]

Supplementary Figure 5. Pareto curves obtained with AI Feynman software. The triangle indicates the knee point.

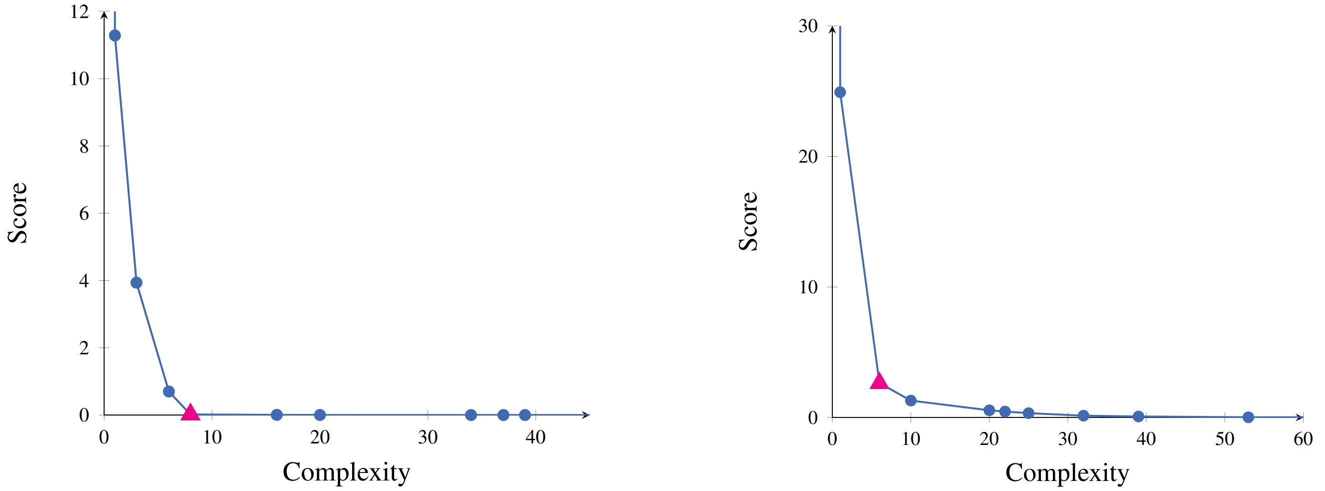

(a) Kepler Solar

(b) Langmuir [5, Table IX]

Supplementary Figure 6. Pareto curves obtained with TuringBot software. The triangle indicates the knee point.

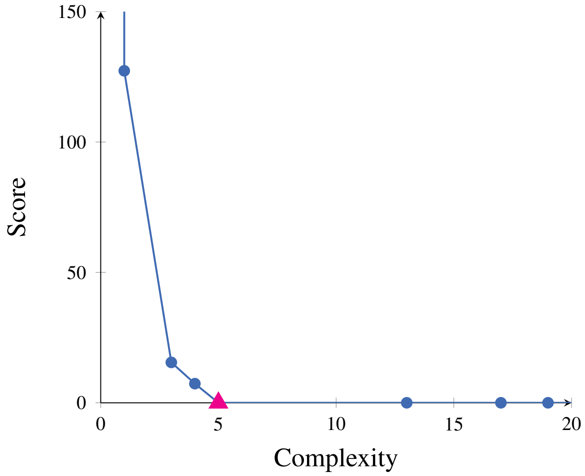

(a) Kepler Solar

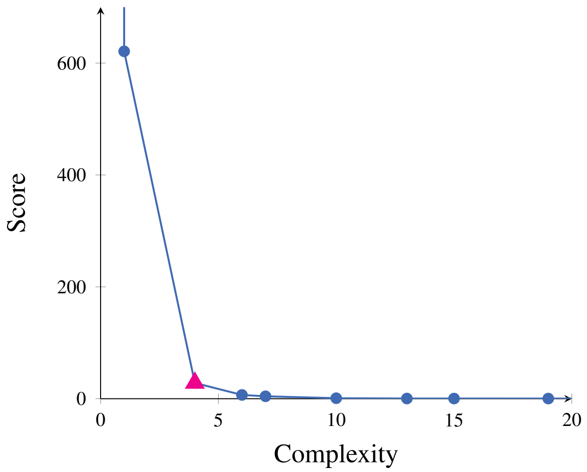

(b) Langmuir [5, Table IX]

Supplementary Figure 7. Pareto curves obtained with PySR software. The triangle indicates what the software identified as best candidate.

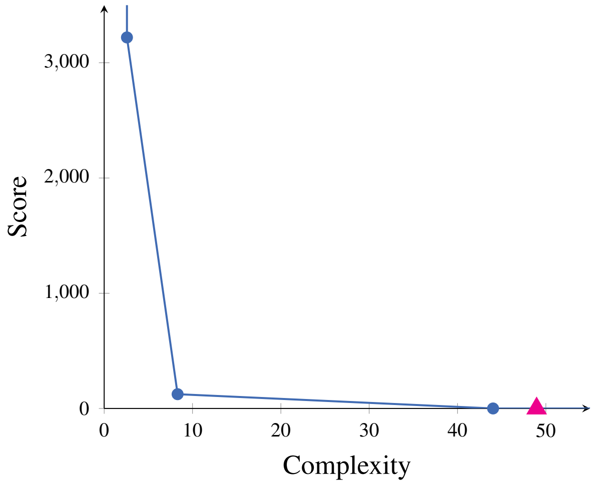

(a) Kepler Solar

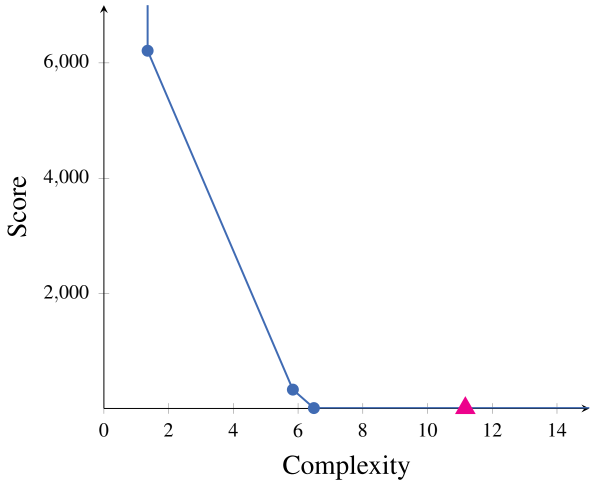

(b) Langmuir [5, Table IX]

Supplementary Figure 8. Pareto curves obtained with BMS software. The triangle indicates what the software identified as best candidate.

Software	Candidate formula $p =$
AI Feynman	$\arccos(-666.0\sin\pi)(0.0+d\sqrt{d})$
	$0.3652d$
	$\mathbf{0.3652}\boldsymbol{d\sqrt{d}}$
	$8.455d\sin(\sqrt{d}/e^{\pi})$
TuringBot	d
	$1.801d$
	$0.8373\ln\Gamma(d)$
	$\mathbf{0.3632}\boldsymbol{d\sqrt{d}}$
	$d\sqrt{0.1318d-(-0.006766\cos(d))}$
	$d\sqrt{0.1316d+0.006946(\cos(d)+0.8752)}-0.009080$
	$(\sqrt{0.01623+d}/2.755)\times$ $(0.02646\cos(-0.9837(0.3384+d))\tanh(-0.3400+0.1561m_2)+d)$
	$(\sqrt{0.01554+d}/2.755)\times$ $(0.02624\cos(-0.9832(0.3700+d))\tanh(-0.2725+\text{round}(0.2006m_2))+d)$
	$(\sqrt{0.01515+d}/2.755)\times$ $(0.02625\cos(-0.9835(0.3613+d))\tanh(-0.2965+\text{round}(0.1732m_2))+d)+$ 0.0002095
PySR	d
	$d/0.5553$
	$0.06912d^2$
	$\boldsymbol{\sqrt{\lvert 0.1319d^3\rvert}}$
	$d\sqrt{\lvert d/(-7.583+(\cos(d)-0.3240)0.02758)\rvert}$
	$d\sqrt{\lvert (d+0.006866)/(-7.583+(\cos(d)-0.3240/0.8472)0.02758)\rvert}$
	$d\sqrt{\lvert (d+0.006866)/(-7.583+(\cos(d+0.006866)-0.3240/0.8472)0.02758)\rvert}$
Bayesian	13.35
	1.342^2d
	$(d/(1.961m_1))^{1.499}$
	$\boldsymbol{(0.5099d)^{1.499}}$

Supplementary Table 7. Pareto front expressions for the Kepler solar dataset. Shown are fronts for the codes AI Feynman, TuringBot, PySR, and Bayesian Machine Scientist. The best candidate solution for each system is marked with bold font.

Software	Candidate formula $p =$
AI Feynman	$\sqrt{0}(-666.0\sin\pi)(-666.0+\sin\pi)$
	$\mathbf{arcsin(0.0003290+\boldsymbol{d}/(\boldsymbol{m_1}/\boldsymbol{d}+1+1))}$
	$\arcsin(-0.3644d(-\sqrt{d/m_1}))$
TuringBot	d
	$1.801d$
	$0.04182d(d+17.79)$
	$\mathbf{0.3632\boldsymbol{d}\sqrt{\boldsymbol{d}}}$
	$d\sqrt{0.1319d/m_1}$
	$1.724d\sqrt{(0.04435(d+0.05064m_1\cos(d))/m_1)}$
	$1.724d\sqrt{0.04433(d+0.04998m_1(\cos(d)+0.2986))/m_1}$
	$1.724d\times$ $\sqrt{0.04431(d+0.05476(0.9761 \mod (-0.06163+d))(\cos(d)+0.4476))/m_1}$
	$1.724d\sqrt{A}$, where $A=0.04432/m_1\times$ $(d+0.05497(1.002 \mod (-0.2991+d))m_1(\cos(d+0.05343)+0.4768))$
	$1.724d\sqrt{A}$, where $A=0.04431/m_1\times$ $(d+0.05434(1.012 \mod (-0.3237+d))(m_1+m_1-1.003)\times$ $(\cos((d+0.04387)/\tanh(1.139d))+0.4895))$
PySR	0.03727
	d^2
	$(\sin(d))^2$
	$\sqrt{\lvert 0.3989d^3\rvert}$
	$\mathbf{\sqrt{\lvert -0.1309\boldsymbol{d}^3/\boldsymbol{m_1}\rvert}}$
	$\sqrt{\lvert -0.1309d^3/(m_1+m_2^2)\rvert}$
	$\sqrt{\lvert -0.1255d^3/m_1\rvert}(1.035-m_2)$
	$\sqrt{\lvert \sin(\sin(-0.4373d^3/(-m_1/0.5873)(m_2+-0.5244)))\rvert}$
	$\sqrt{\lvert -0.4373d^3/(m_1/-0.5873)(m_2d/sin(d)-0.5244)\rvert}$
Bayesian	m_2
	$0.4825(-0.02983+\sin(d))$
	$(-0.3064^3+d)0.4613$
	$(d^2+0.3411d)0.5240$
	$d(0.1650+d/(m_1/0.1815))$
	$\mathbf{0.3641\boldsymbol{m_1}^{-0.49281}\boldsymbol{d}^{(4.0981+\boldsymbol{m_2})0.3641}}$

Supplementary Table 8. Pareto front expressions for the Kepler exoplanet dataset. Shown are the fronts returned by the codes AI Feynman, TuringBot, PySR, and Bayesian Machine Scientist. The best candidate solution for each system is marked in bold font.

Software	Candidate formula $p =$
AI Feynman	$1/(666.0+\pi/\sin\pi)$
	$\boldsymbol{\arcsin(1.282+(\cos(\cos((\pi-\sqrt{m_2})/d))+m_1)\times \sin(e^{(\sin(\cos(\cos((\pi-\sqrt{m_2})/d))+m_1+\cos(\cos((\pi-\sqrt{m_2})/d))+m_1))^{-1}}))}$
PySR	709.4
	$d/0.1362$
	$\boldsymbol{\sqrt{\lvert d^3/(m_1+m_2)\rvert}}$
	$\sqrt{\lvert d^3/(m_1+m_2)\rvert}+m_1$
	$\sqrt{\lvert -0.9276d^3/(0.8620m_1+m_2)\rvert}$
	$\sqrt{\lvert (d+0.2389)^3/1.096/(m_2+0.8451m_1)\rvert}$
	$\sqrt{\lvert (d+0.2070)^3/1.093/\lvert m_2+0.8485m_1\rvert\rvert}$
	$0.9852\sqrt{\lvert d^3/(m_1+\sqrt{\lvert m_1+-0.3363\rvert})\rvert}-5.460+m_1-5.491$
Bayesian	d
	$4.302((-26.43m_1+d)^{1.035}+d/(m_1m_2))m_2$
	$\boldsymbol{(d(m_2+m_1-0.006465(m_2^3+m_1^{m_2}))^{-0.3340})^{1.499}}$

Supplementary Table 9. Pareto front expressions for the Kepler binary stars dataset. Shown are the fronts returned by the codes AI Feynman, PySR, and Bayesian Machine Scientist. The best candidate solution is marked with bold font.

Software	Candidate formula $y =$
AI Feynman	$\arccos(2.909 + (\cos\pi - 1))$
	$\sqrt{0.04 - 0.02v}$
	$\mathbf{-0.05122v}$
	$-1.018 + \sin(e^{e^{\sin(\sin(v-1-1))}})$
	$-0.02009e^{(\sqrt{v}+\sin((v-1)^{-1}))}$
TuringBot	-2.471
	$-0.1675v$
	$\mathbf{-0.005628v^2}$
	$-0.004998v(3.977 + v)$
	$(3.995 + v)(-0.005008(v - \cos(0.6416v)))$
	$(3.281 + v)(-0.005138(v - 1.385\cos(0.6491v)))$
	$(-0.005488(1.114 + v) - (\cos(2.744v)/(-6.617(-5.780 + v))))v$
	$(-0.005486(1.166 + v) - ((0.033306 + \cos(2.7436v))/(-6.6226(-5.946 + v))))v$
	$(2.008 + v)(-0.005302(v - \tan(1.054\cos(-0.6517v - 0.8977/v) - 0.1687)))$
	$0.005332((-0.3758 - (v-$ $\operatorname{arctanh}(\cos(2.736(-0.2379(0.1817 + v))) - 0.04407)))(1.589 + v) - 2.227/v)$
	$0.005324((-0.3791 - (v - 0.9871\operatorname{arctanh}(\cos(2.735(-0.2378(0.1882 + v)))-$ $0.04432)))(1.641 + v) - (2.404/v - 0.3656))$
	$0.005323((-0.3824 - (v - \operatorname{arctanh}(\cos(2.735(-0.2379(0.1891 + v)))-$ $0.04436)/1.014))(1.643 + v) - (2.425/v - 0.4147))$
	$0.005323((-0.3831 - (v - \operatorname{arctanh}(\cos(-2.735(0.2379(0.1880 + v)))-$ $0.04424)/1.014))(1.643 - 0.001885/(3.914 - v) + v) - (2.436/v - 0.4321))$
PySR	2.471
	$\sqrt{\lvert v\rvert}$
	$\mathbf{(-0.07502v)^2}$
	$(0.07701(v + \sin(v)))^2$
	$(0.07690(v + \sin(1.848v)))^2$
	$(-0.07196(v + e^{\sin(1.811v)}))^2$
	$(0.07388(v - 0.4611 + e^{\sin(1.818(v+0.1042))}))^2$
	$(0.07388(v - 0.5577 + e^{\sin(1.818(v-\sin(-1.277/v)))}))^2$
	$(0.07388(v - 0.5577 + e^{\sin(1.818(v+1.320/(v+1.052)))}))^2$
Bayesian	2.471
	$-(-0.4459)1.081^v$
	$\mathbf{(0.07502v)^2}$

Supplementary Table 10. Pareto front expressions for the time dilation dataset. Shown are the fronts returned by the codes AI Feynman, TuringBot, PySR, and Bayesian Machine Scientist. The best candidate solution for each system is marked with bold font.

Software	Candidate formula $q =$
AI Feynman	$1/0.03000$
	$38.50+p$
	$\mathbf{30.60+p+p}$
	$\arcsin(0.8065\sin(e^{p+p}-p))$
	$27.10(\log(p+p+1+1+1)-1)$
TuringBot	67.01
	$\mathbf{19.45\,arccosh(p)}$
	$(1.570+p)/(0.1128+0.008879p)$
	$(\cos(\mathrm{round}(-9.038p))+19.94)\,\mathrm{arcsinh}(\lfloor p\rfloor)$
	$(\cos(\mathrm{round}(-9.038p))+19.90)\,\mathrm{arcsinh}(\lfloor p\rfloor+0.09014)$
	$(\cos(\mathrm{round}(-9.038p))+19.86)\,\mathrm{arcsinh}(\lfloor 1.014p\rfloor+0.1024)$
	$5.349(0.4604-\cos(0.09345(\tan(-0.1359p)-1.649-\lceil p\rceil)))+\mathrm{arcsinh}(p)/0.05244$
	$5.425(0.5252-\cos(0.09347(0.7537(\mathrm{round}(\tan(-0.1360(p-0.38043)))-2.036)-\lceil p\rceil)))+\mathrm{arcsinh}(p)/0.05269$
	$19.74\,\mathrm{arccosh}(-0.3987+p)+\sqrt{0.8632(p+1.820)}\times\cos(-0.7427(4.618/(\tan(0.06620+p)+p-1.738\cos(p))-(-0.8143+\mathrm{round}(p))))$
PySR	67.01
	$\mathbf{23.50\log(\lvert p\rvert)}$
	$20.07(\log(\lvert p\rvert)+0.5671)$
	$65.87\log(\lvert\log(\lvert p+1.991\rvert)\rvert)$
	$66.09\log(\lvert\log(\lvert p+\log(\lvert 11.97-p\rvert)\rvert)\rvert)$
	$19.54(\log(\lvert p\rvert)+\cos(\sin(\log(\lvert(p-8.270)/0.8487\rvert))))$
	$19.54(\log(\lvert p\rvert)+\cos(\sin(\log(\lvert-1.175(p-8.270+0.1542)\rvert))))$
	$19.53(\log(\lvert p\rvert)+\cos(\sin(\log(\lvert(\lvert p-8.320/1.048\rvert+0.2113)/20.07\rvert))))$
Bayesian	67.01
	$(200865.8p)^{0.2770}$
	$((12.71p+19.97)/0.1128)/(p+12.71)$
	$\mathbf{(112.6p+177.0)/(p+12.71)}$

Supplementary Table 11. Pareto front expressions for the Langmuir [5, Table IX] dataset. Shown are the fronts returned by the codes AI Feynman, TuringBot, PySR, and Bayesian Machine Scientist. The best candidate solution for each system is marked with bold font.

Software	Candidate formula $q =$
AI Feynman	$(-666.0\sin\pi)^2$
	$\arcsin(0.03p+0.72)$
	$\mathbf{0.06}\boldsymbol{p}\mathbf{+0.79}$
	$-0.1042+\sqrt{\sqrt{\sqrt{\sqrt{p}}}}$
	$1.615\sin(\log(\log(p+\pi+1+1)))$
TuringBot	1.155
	$p^{0.1177}$
	$\boldsymbol{p/(p+8.057)+0.7518}$
	$1.615-0.9128^{2.6189+p-0.1221/p}$
	$\arctan(0.8093+0.1852p)+0.1238-0.006844/(\tan(p)-0.09319p)$
	$\arctan(0.7984+0.1826p)+0.1258-0.004073/(\tan(p)-0.09197p-0.02751)$
	$0.001026(-24.31+p)+$ $\arctan((18.68(-0.06174+p))^{\log(1.054+0.02370(\cos(-1.124(-0.7298+p))+p))})$
	$0.001052(-25.27+p)+$ $\arctan((17.83(-0.05962+p))^{\log(1.056+0.02719(\cos(1.125(-0.6507+p))+0.8783p))})$
	$0.001067((0.4667-0.01580p)\tan(p)-24.73+p)+$ $\arctan((16.71(-0.05784+p))^{\log(1.059+0.02732(\cos(1.087(-0.5273+p))+0.8647p))})$
	$0.001057(\cos(p)+(0.4217-0.01331p)\tan(p)-24.46+p)+$ $\arctan((16.09(-0.05778+p))^{\log(1.060+0.02710(\cos((-0.5464+p)/(-0.9153))+0.8754p))})$
PySR	1.155
	$\boldsymbol{\sqrt{\lvert\sqrt{\lvert\sqrt{\lvert p\rvert}\rvert}\rvert}}$
	$\sqrt{\lvert\sqrt{\lvert\sqrt{\lvert p\rvert}\rvert}\rvert}-0.05434$
	$\sqrt{\lvert\sqrt{\lvert\sqrt{\lvert p\rvert}-\sin(0.3785p)\rvert}\rvert}$
	$\sqrt{\lvert\sqrt{\lvert\sqrt{\lvert p\rvert}-(\sin(\log(\lvert p+0.9549\rvert)))^2\rvert}\rvert}$
	$\sqrt{\lvert\sqrt{\lvert\sqrt{\lvert p\rvert}-(\sin(\sqrt{\lvert p/1.495\rvert}))^3-(0.01481p)^2\rvert}\rvert}$
	$\sqrt{\lvert\sqrt{\lvert\sqrt{\lvert p\rvert}-(\sin(\sqrt{\lvert p/-1.413\rvert}))^3/1.145-(-0.01478p)^2\rvert}\rvert}$
Bayesian	1.155
	$\boldsymbol{p^{0.1177}}$

Supplementary Table 12. Pareto front expressions for the Sun et al. [6, Table 1] dataset. Shown are the fronts returned by the codes AI Feynman, TuringBot, PySR, and Bayesian Machine Scientist. The best candidate solution for each system is marked with bold font.

In Supplementary Table 13, we show the results for AI-Descartes, AI Feynman, PySR and BMS on 81 problems (problems that do not contain trigonometric functions) from the FSRD (Feynman Symbolic Regression Database) using our version of the data (fewer data point and with noise) for each problem.

For each solver, we check if the list of output solutions contains the true formula, which is indicated by a ✓in the appropriate column. If the functional form is correct but has a single multiplicative constant that is slightly different from the true constant, we indicate this by $\checkmark^1$. As an example, instead of obtaining $q_2 E_f$ for I.12.5, we obtain $0.99679 q_2 E_f$ as a potential solution. If a constant that is added to other terms is slightly incorrect, we indicate this by $\checkmark^2$. For example, PySR obtains $p_F V/(\gamma - 1.0014104)$ instead of $p_F V/(\gamma - 1)$ as a solution for problem I.39.11. Finally, if a formula is correct other than for a slightly erroneous power, we indicate this by a $\checkmark^3$; for example, in the solution returned by Bayesian Machine Scientist to II.3.24, the power of r is 1.98285 instead of 2. It is clear that all of the solvers struggle with many of the problems, especially those that are not a simple ratio of monomials. Thus, our version of FSRD dataset - which is designed to mimic real-life data in that we have both few data points and a nontrivial amount of noise - is quite nontrivial to deal with. Our tool, AI-Descartes, is the best performing, achieving an accuracy of 60.49%, obtaining the correct solution for 49 out of 81 problems as a candidate solution modulo a small error in a multiplicative constant or in an addition, compared to 40.74% for AI Feynman, 49.38% for PySR, and 48.15% for Bayesian Machine Scientist (BMS). Therefore, our method outperform the next best solver (PySR) with an improvement of 11.11%. This gap in performance is the reason why we choose to use our symbolic regression engine instead of other methods.

In Supplementary Table 14, we provide the results on a subset of 15 problems from the ones used in Supplementary Table 13, to compare with TuringBot. We determined this subset to comply with the requirements of the freely available version of TuringBot which allows a maximum of 2 variables per problem. Also in this subset of problems, we outperform all the state-of-the-art methods. However, the performance of the tools is generally higher in Supplementary Table 14, compared to Supplementary Table 13, since these selected 15 problems are much simpler and thus easier to solve.

Finally, we manually extracted the background theory of 5 problems from Feynman's Lectures (I.27.6, I.34.8, I.43.16, II.10.9 and II.34.2). We were able to execute the full AI-Descartes pipeline: The results are provided in Supplementary Table 15. For all of these 5 problems the SR module is able to induce the right formula (as showed in Supplementary Table 13) and the Reasoning module is able to derive it, either directly or with existential quantification over the numerical constants. This result shows that for some problems from FSRD, the reasoning module is able to successfully identify the correct formula from a set of candidates with comparable errors over the data.

In conclusion, the tools we compared against are unable to consistently identify the correct formula on the six datasets (obtained from real experiments) considered in this work, or on the 81 synthetic datasets derived from the Feynman Symbolic Regression Database. Although some of them produce the correct formula among the top candidates, they do not have a principled way to identify it from among the list of candidates.

Label	Formula	AI-Descartes	AI Feynman	PySR	BMS
I.6.20a	$e^{-\theta^2/2}/\sqrt{2\pi}$	X	X	X	X
I.6.20	$e^{-\frac{\theta^2}{2\sigma^2}}/\sqrt{2\pi\sigma^2}$	X	X	X	X
I.6.20b	$e^{-\frac{(\theta-\theta_1)^2}{2\sigma^2}}/\sqrt{2\pi\sigma^2}$	X	X	X	X
I.8.14	$\sqrt{(x_2-x_1)^2+(y_2-y_1)^2}$	X	X	X	X
I.9.18	$\frac{Gm_1m_2}{(x_2-x_1)^2+(y_2-y_1)^2+(z_2-z_1)^2}$	X	X	X	X
I.10.7	$\frac{m_0}{\sqrt{1-v^2/c^2}}$	✓	X	X	X
I.11.19	$x_1y_1+x_2y_2+x_3y_3$	X	X	X	X
I.12.1	μN_n	✓	✓[2]	✓	✓
I.12.2	$q_1q_2/(4\pi\varepsilon r^2)$	✓[1]	X	✓[1]	✓[1]
I.12.4	$q_1/(4\pi\varepsilon r^2)$	✓[1]	✓[1]	✓[1]	✓[1]
I.12.5	q_2E_f	✓[1]	✓[2]	✓	✓
I.13.4	$\frac{1}{2}m(v^2+u^2+w^2)$	X	X	X	X
I.13.12	$Gm_1m_2(\frac{1}{r_2}-\frac{1}{r_1})$	X	X	X	X
I.14.3	mgz	✓	✓[1]	✓	✓
I.14.4	$k_{spring}x^2/2$	✓[1]	✓[1]	✓[1]	✓[1]
I.15.3x	$\frac{x-ut}{\sqrt{1-u^2/c^2}}$	X	X	X	X
I.15.3t	$\frac{t-ux/c^2}{\sqrt{1-u^2/c^2}}$	X	X	X	X
I.15.10	$\frac{m_0v}{\sqrt{1-v^2/c^2}}$	✓[1]	X	X	X
I.16.6	$\frac{u+v}{1+uv/c^2}$	✓[2]	X	X	X
I.18.4	$\frac{m_1r_1+m_2r_2}{m_1+m_2}$	✓	X	X	X
I.24.6	$\frac{1}{4}m(\omega^2+\omega_0^2)x^2$	X	X	X	X
I.25.13	q/C	✓	✓[1]	✓	✓
I.27.6	$1/(\frac{1}{d_1}+\frac{n}{d_2})$	✓[1]	✓[2]	✓	X
I.29.4	$\frac{\omega}{C}$	✓	✓[2]	✓	✓
I.32.5	$q^2a^2/(6\pi\varepsilon c^3)$	X	X	X	X
I.32.17	$(\frac{1}{2}\varepsilon cE_f^2)(\frac{8\pi r^2}{3})(\frac{\omega^4}{(\omega^2-\omega_0^2)^2})$	X	X	X	X
I.34.8	qvB/p	✓	✓[1]	✓	✓[1]
I.34.10	$\omega_0/(1-v/c)$	✓	✓[1]	✓	X
I.34.14	$\frac{1+v/c}{1-v^2/c^2}\omega_0$	X	X	X	X
I.34.27	$\frac{h\omega}{2\pi}$	✓[1]	✓[1]	✓[1]	✓[1]
I.38.12	$4\pi\varepsilon h^2/(mq^2)$	✓[1]		✓[1]	✓[2]
I.39.10	$\frac{3}{2}p_FV$	✓[1]	✓[1]	✓[1]	✓[1]
I.39.11	$\frac{1}{\gamma-1}p_FV$	✓[2]	✓[1]	✓[2]	✓[2]
I.39.22	nk_bT/V	✓	✓[1]	✓	✓
I.40.1	$n_0e^{-\frac{mgx}{k_bT}}$	X	X	X	X
I.41.16	$\frac{h\omega^3}{\pi^2c^2(e^{\frac{h\omega}{k_bT}}-1)}$	X	X	X	X
I.43.16	$\mu_{drift}qV_e/d$	✓[1]	✓[2]	✓	✓
I.43.31	μ_ek_bT	✓	✓[1]	✓	✓[2]
I.43.43	$\frac{k_bv}{(\gamma-1)A}$	X	✓[1]	X	X
I.44.4	$nk_bT\ln\frac{V2}{V1}$	X	X	X	X
I.47.23	$\sqrt{\frac{\gamma pr}{\rho}}$	✓[2]	✓[1]	✓	✓[2]
I.48.20	$\frac{mc^2}{\sqrt{1-v^2/c^2}}$	✓	X	X	X
II.2.42	$k(T_2-T_1)A/d$	X	X	✓	X
II.3.24	$P/(4\pi r^2)$	✓[1]	✓[1]	✓[1]	✓[3]

II.4.23	$q/(4\pi\epsilon r)$	$\checkmark^1$	$\checkmark^1$	$\checkmark^1$	$\checkmark^1$
II.6.15a	$\frac{3}{4\pi\epsilon}\frac{p_d z}{r^5}\sqrt{x^2+y^2}$	X	X	X	X
II.8.7	$\frac{3}{5}\frac{q^2}{4\pi\epsilon d}$	$\checkmark^1$	$\checkmark^1$	$\checkmark^1$	$\checkmark^2$
II.8.31	$\epsilon E_f^2/2$	$\checkmark^1$	$\checkmark^1$	$\checkmark^1$	$\checkmark^1$
II.10.9	$\frac{\sigma_{den}}{\epsilon}\frac{1}{1+\chi}$	$\checkmark^1$	$\checkmark^2$	$\checkmark^2$	$\checkmark^2$
II.11.3	$\frac{qE_f}{m(\omega_0^2-\omega^2)}$	X	X	X	X
II.11.20	$n_\rho p_d^2 E_f/(3k_bT)$	$\checkmark^1$	X	X	$\checkmark^2$
II.11.27	$\frac{n\alpha}{1-n\alpha/3}\epsilon E_f$	$\checkmark^1$	X	X	X
II.11.28	$1+\frac{n\alpha}{1-n\alpha/3}$	$\checkmark^1$	X	X	X
II.13.17	$\frac{1}{4\pi\epsilon c^2}\frac{2I}{r}$	$\checkmark^1$	X	$\checkmark^1$	X
II.13.23	$\frac{\rho_{c_0}}{\sqrt{1-v^2/c^2}}$	$\checkmark^1$	X	X	X
II.13.34	$\frac{\rho_{c_0}v}{\sqrt{1-v^2/c^2}}$	X	X	X	X
II.21.32	$\frac{q}{4\pi\epsilon r(1-v/c)}$	X	X	X	X
II.24.17	$\sqrt{\frac{\omega^2}{c^2}-\frac{\pi^2}{d^2}}$	X	X	X	X
II.27.16	$\epsilon c E_f^2$	$\checkmark$	X	$\checkmark$	$\checkmark$
II.27.18	ϵE_f^2	$\checkmark^1$	$\checkmark^1$	$\checkmark$	$\checkmark$
II.34.2a	$qv/(2\pi r)$	$\checkmark^1$	$\checkmark^1$	$\checkmark^1$	$\checkmark^1$
II.34.2	$qvr/2$	$\checkmark^1$	$\checkmark^1$	$\checkmark^1$	$\checkmark^1$
II.34.11	$gqB/(2m)$	$\checkmark$	$\checkmark^1$	$\checkmark^1$	$\checkmark^1$
II.34.29a	$qh/(4\pi m)$	$\checkmark^1$	$\checkmark^1$	$\checkmark^1$	$\checkmark^1$
II.34.29b	$g\mu_M BJ_z/h$	$\checkmark^2$	X	$\checkmark^1$	$\checkmark^1$
II.35.18	$\frac{n_0}{e^{\frac{mom\times B}{kb\times T}}+e^{\frac{-mom\times B}{(kb\times T)}}}$	X	X	X	X
II.36.38	$\frac{\mu_m B}{k_bT}+\frac{\mu_m\alpha M}{\epsilon c^2 k_bT}$	X	X	X	X
II.37.1	$\mu_M(1+\chi)B$	$\checkmark^1$	X	$\checkmark^1$	$\checkmark^2$
II.38.3	YAx/d	$\checkmark^1$	$\checkmark^2$	$\checkmark$	$\checkmark^2$
II.38.14	$\frac{Y}{2(1+\sigma)}$	$\checkmark^1$	$\checkmark^2$	X	$\checkmark^2$
III.4.32	$1/(e^{\frac{h\omega}{k_bT}}-1)$	X	X	X	X
III.4.33	$h\omega/(e^{\frac{h\omega}{k_bT}}-1)$	X	X	X	X
III.7.38	$2\mu_M B/h$	$\checkmark^1$		$\checkmark^1$	$\checkmark^1$
III.10.19	$\mu_M\sqrt{B_x^2+B_y^2+B_z^2}$	X	X	X	X
III.12.43	nh	$\checkmark^1$	$\checkmark^1$	$\checkmark^1$	$\checkmark^1$
III.13.18	$2Ed^2k/h$	$\checkmark^1$	X	X	$\checkmark^1$
III.14.14	$I_0(e^{\frac{qV_e}{k_bT}}-1)$	X	X	X	X
III.15.14	$h^2/(2Ed^2)$	X	X	$\checkmark^1$	$\checkmark^2$
III.15.27	$2\pi\alpha/(nd)$	$\checkmark^1$	$\checkmark^1$	$\checkmark^1$	$\checkmark^1$
III.19.51	$\frac{-mq^4}{2(4\pi\epsilon)^2h^2}\frac{1}{n^2}$	X	X	X	X
III.21.20	$-\rho_{c_0}qA_{vec}/m$	$\checkmark^1$	$\checkmark^1$	$\checkmark$	$\checkmark$
		**AI-Descartes**	**AI Feynman**	**PySR**	**BMS**
Number of ($\checkmark$, $\checkmark^1$, $\checkmark^2$, $\checkmark^3$, X)		(13, 32, 4, 0, 32)	(0, 25, 8, 0, 48)	(16, 21, 2, 0, 41)	(10, 17, 11, 1, 42)
Total $\checkmark^*$		49/81	33/81	40/81	39/81
**Accuracy**		**60.49%**	40.74%	49.38%	48.15%

Supplementary Table 13. Results on 81/100 problems from the Feynman Database for Symbolic Regression (problems not containing trigonometric functions). The accuracy of the best method is marked with bold font.

Label	AI-Descartes	AI Feynman	PySR	BMS	TuringBot
I.6.20a	X	X	X	X	X
I.6.20	X	X	X	X	X
I.12.1	✓	$✓^2$	✓	✓	✓
I.12.5	$✓^1$	$✓^2$	✓	✓	✓
I.14.4	$✓^1$	$✓^1$	$✓^1$	$✓^1$	$✓^1$
I.25.13	✓	$✓^1$	✓	✓	✓
I.29.4	✓	$✓^2$	✓	✓	✓
I.34.27	$✓^1$	$✓^1$	$✓^1$	$✓^1$	$✓^1$
I.39.10	$✓^1$	$✓^1$	$✓^1$	$✓^1$	$✓^1$
II.3.24	$✓^1$	$✓^1$	$✓^1$	$✓^3$	$✓^1$
II.8.31	$✓^1$	$✓^1$	$✓^1$	$✓^1$	$✓^1$
II.11.28	$✓^1$	X	X	X	X
II.27.18	$✓^1$	$✓^1$	✓	✓	✓
II.38.14	$✓^1$	$✓^2$	X	$✓^2$	$✓^2$
III.12.43	$✓^1$	$✓^1$	$✓^1$	$✓^1$	$✓^1$
Number of (✓, $✓^1$, $✓^2$, $✓^3$, X)	(3, 10, 0, 0, 2)	(0, 8, 4, 0, 3)	(5, 6, 0, 0, 4)	(5, 5, 1, 1, 3)	(5, 6, 1, 0, 3)
Total $✓^*$	13/15	12/15	11/15	12/15	12/15
Accuracy	**86.67%**	80%	73.33%	80%	80%

Supplementary Table 14. Results from running TuringBot on 15 problems from the Feynman Database for Symbolic Regression with up to two variables. The performance is higher compared to Supplementary Table 13 since problems with at most 2 variables are easier to solve. The accuracy of the best method is marked with bold font.

Label	Ground truth Formula	AI-Descartes formula (SR)	Derivability	∃-Derivability
I.27.6	$1/(\frac{1}{d_1}+\frac{n}{d_2})$	$\frac{0.9979 d_1^{-1} n^{-2}}{d_1^{-2} n^{-2} + d_1^{-1} d_2^{-1} n^{-1}}$	X	✓
I.34.8	qvB/p	$qvBp^{-1}$	✓	n/a
I.43.16	$\mu_d q V_e/d$	$0.9993 q \mu_d V_e d^{-1}$	X	✓
II.10.9	$\frac{\sigma_{den}}{\varepsilon(1+\chi)}$	$\frac{0.9960\sigma_{den}}{\varepsilon+\varepsilon\chi}$	X	✓
II.34.2	$qvr/2$	$0.5022qvr$	X	✓

Supplementary Table 15. Results from running the AI-Descartes' Reasoning module on 5 selected problems (with available background theory) from the Feynman Database for Symbolic Regression. We are able to prove (either directly or existentially quantifying over the numerical constants) the correct formula, which is a part of the output of the SR module, for all the problems.

Method	Configurations
AI Feynman	NN_epochs=500<br>All other parameters=default
TuringBot	Time limit (s)=120<br>binary_operators=["*", "/", "+", "pow", "fmod"]<br>unary_operators=["sin", "cos", "tan", "arcsin", "arccos", "arctan", "exp", "log", "log2", "sqrt", "sinh", "cosh", "tanh", "arcsinh", "arccosh", "arctanh", "abs", "floor", "ceil", "round", "sign", "tgamma", "lgamma", "er"]<br>Search metric="RMS error"
PySR	niterations=300<br>binary_operators=["*", "/", "+", "-", "div", "mult", "plus", "sub"]<br>unary_operators=["sin", "cos", "exp", "sqrt", "square", "cube", "log", "neg", "abs"]<br>model_selection="best"
Bayesian M.S.	kepler solar: ./Prior/final_prior_param_sq.named_equations.nv3.np3.2017-06-13 08:55:24.082204.dat<br>kepler exo: ./Prior/final_prior_param_sq.named_equations.nv3.np3.2017-06-13 08:55:24.082204.dat<br>kepler binary: ./Prior/final_prior_param_sq.named_equations.nv3.np3.2017-06-13 08:55:24.082204.dat<br>langmuir Sun: ./Prior/final_prior_param_sq.named_equations.nv1.np3.2017-10-18 18:07:35.262530.dat<br>langmuir tab: ./Prior/final_prior_param_sq.named_equations.nv1.np3.2017-10-18 18:07:35.262530.dat<br>relativity: ./Prior/final_prior_param_sq.named_equations.nv1.np3.2017-10-18 18:07:35.262530.dat

Supplementary Table 16. Configuration parameters used for comparing with AI Feynman, TuringBot, PySR and Bayesian Machine Scientist.

Supplementary References

1. NASA. Planets Factsheet. https://nssdc.gsfc.nasa.gov/planetary/factsheet/ (2017).
2. NASA. Exoplanet Archive. https://exoplanetarchive.ipac.caltech.edu/) (2017).
3. Novaković, B. Orbits of five visual binary stars. *Balt. Astron.* **16**, 435–442 (2007).
4. Chou, C. W., Hume, D. B., Rosenband, T. & Wineland, D. J. Optical clocks and relativity. *Science* **329**, 1630–1632 (2010).
5. Langmuir, I. The adsorption of gases on plane surfaces of glass, mica and platinum. *J. Amer. Chem. Soc.* **40**, 1361–1403 (1918).
6. Sun, M. S., Shah, D. B., Xu, H. H. & Talu, O. Adsorption equilibria of C1 to C4 alkanes, CO2, and SF6 on silicalite. *J. Phys. Chem.* **102**, 1466–1473 (1998).
7. Talu, O. & Myers, A. L. Rigorous thermodynamic treatment of gas adsorption. *AIChE J.* **34**, 1887–1893 (1988).
8. Schmidt, M. & Lipson, H. *Eureqa* (Nutonian, Somerville, USA, 2014). (Version 0.98 beta) [Software], available from www.nutonian.com.
9. Schmidt, M. & Lipson, H. Distilling free-form natural laws from experimental data. *Science* **324**, 81–85 (2009).
10. Stephens, T. Genetic programming in python, with a scikit-learn inspired api: gplearn. https://gplearn.readthedocs.io/en/stable/ (2019). Version 0.41.
11. Korns, M. F. Accuracy in symbolic regression. In Riolo, R., Vladislavleva, E. & Moore, J. H. (eds.) *Genetic Programming Theory and Practice IX*, 129—151 (Springer, Berlin, 2011).
12. Cozad, A. *Data- and theory-driven techniques for surrogate-based optimization*. Ph.D. thesis, Carnegie Mellon, Pittsburgh, PA (2014).
13. Avron, H., Horesh, L., Liberti, L. & Nahamoo, D. Globally convergent system and method for automated model discovery (2015). U.S. Patent Application 20170004231, application No. 14/755,942 filed June 30, 2015.
14. Austel, V. *et al.* Globally optimal symbolic regression. *NIPS Symp. on Interpret. Mach. Learn.* (2017).
15. Cozad, A. & Sahinidis, N. V. A global MINLP approach to symbolic regression. *Math. Program., Ser. B* **170**, 97–119 (2018).
16. Sahinidis, N. V. Baron: A general purpose global optimization software package. *J. Glob. Optim.* **8**, 201—205 (1996).
17. Maher, S. J. *et al.* The SCIP optimization suite 4.0. Tech. Rep. 17-12, ZIB, Takustr.7, 14195 Berlin (2017).
18. Izzo, D., Biscani, F. & Mereta, A. Differentiable genetic programming. In et al., J. M. (ed.) *EuroGP 2017, LNCS 10196*, 35—51 (2017).
19. Bergmann, M., Moor, J. & Nelson, J. *The Logic Book* (McGraw-Hill Higher Education, 2013).
20. Enderton, H. & Enderton, H. *A Mathematical Introduction to Logic* (Elsevier Science, 2001).
21. Meurer, A. *et al.* SymPy: symbolic computing in Python. *PeerJ Comput. Sci.* **3**, e103 (2017). Version: 1.5.1, https://www.sympy.org.
22. SWI-prolog. https://www.swi-prolog.org. Version: 8.3.3.
23. Wolfram Mathematica. https://www.wolfram.com. Version: 12.
24. Baumgartner, P., Bax, J. & Waldmann, U. Beagle – A Hierarchic Superposition Theorem Prover. In Felty, A. P. & Middeldorp, A. (eds.) *CADE-25 – 25th International Conference on Automated Deduction*, vol. 9195 of *LNAI*, 367–377 (2015).
25. Fulton, N., Mitsch, S., Quesel, J.-D., Völp, M. & Platzer, A. KeYmaera X: An axiomatic tactical theorem prover for hybrid systems. In *Lecture Notes in Computer Science*, vol. 9195 (2015).

26. KeYmaera X. http://www.ls.cs.cmu.edu/KeYmaeraX/. Version: 4.8.

27. Platzer, A. Differential dynamic logic for hybrid systems. *J. Autom. Reas.* (2008).

28. Udrescu, S.-M. & Tegmark, M. AI Feynman: A physics-inspired method for symbolic regression. *Sci. Adv.* (2020).

29. Kitano, H. Artificial intelligence to win the Nobel prize and beyond: Creating the engine for scientific discovery. *AI Mag.* **37**, 39–49 (2016).

30. Martius, G. & Lampert, C. H. Extrapolation and learning equations. In *Proceedings of the 29th Conference on Neural Information Processing Systems (NIPS-16)* (2016).

31. Lample, G. & Charton, F. Deep learning for symbolic mathematics. *arXiv:* **1912.01412** (2019).

32. Guss, W. H. & Salakhutdinov, R. On universal approximation by neural networks with uniform guarantees on approximation of infinite dimensional maps. *arXiv:* **abs/1910.01545** (2019).

33. Arabshahi, F., Singh, S. & Anandkumar, A. Combining symbolic expressions and black-box function evaluations in neural programs. In *ICLR* (2018).

34. Goeßmann, A. *et al.* Tensor network approaches for learning non-linear dynamical laws. In *First Workshop on Quantum Tensor Networks in Machine Learning, 34th Conference on Neural Information Processing Systems (NeurIPS 2020)* (2020).

35. Iten, R., Metger, T., Wilming, H., Rio, L. & Renner, R. Discovering physical concepts with neural networks. *Phys. Rev. Lett.* **124** (2020).

36. Cranmer, M. *et al.* Discovering symbolic models from deep learning with inductive biases. In *NeurIPS 2020: Proceedings of the 34th International Conference on Neural Information Processing Systems* (2020).

37. Derner, E., Kubalík, J., Ancona, N. & Babuska, R. Symbolic regression for constructing analytic models in reinforcement learning. *arXiv:* **1903.11483** (2019).

38. Udrescu, S. *et al.* AI Feynman 2.0: Pareto-optimal symbolic regression exploiting graph modularity. In Larochelle, H., Ranzato, M., Hadsell, R., Balcan, M. & Lin, H. (eds.) *Advances in Neural Information Processing Systems 33: Annual Conference on Neural Information Processing Systems 2020, NeurIPS 2020, December 6-12, 2020, virtual* (2020).

39. Jin, Y., Fu, W., Kang, J., Guo, J. & Guo, J. Bayesian symbolic regression. *arXiv[Methodology]:* **1910.08892** (2019).

40. Lee, D., Szegedy, C., Rabe, M. N., Loos, S. M. & Bansal, K. Mathematical reasoning in latent space. *arXiv:* **1909.11851** (2019).

41. Castelvecchi, D. AI Copernicus 'discovers' that earth orbits the sun. *Nature* **575**, 266–267 (2019).

42. Ellis, K. *et al.* Dreamcoder: Bootstrapping inductive program synthesis with wake-sleep library learning. *Assoc. for Comput. Mach.* 835–850 (2021).

43. Guimerà, R. *et al.* A Bayesian machine scientist to aid in the solution of challenging scientific problems. *Sci. Adv.* **6** (2020).

44. Munafo, R. RIES - Find algebraic equations, given their solution. https://mrob.com/pub/ries/.

45. Brunton, S. L., Proctor, J. L. & Kutz, J. N. Discovering governing equations from data by sparse identification of nonlinear dynamical systems. *Proc. Natl. Acad. Sci.* **113**, 3932–3937 (2016).

46. Ouyang, R., Curtarolo, S., Ahmetcik, E., Scheffler, M. & Ghiringhelli, L. SISSO: a compressed-sensing method for identifying the best low-dimensional descriptor in an immensity of offered candidates. *Phys. Rev. Mater.* **2(08)** (2018).

47. Scott, J., Panju, M. & Ganesh, V. LGML: Logic Guided Machine Learning. *arXiv:* **2006.03626** (2021).

48. Ashok, D., Scott, J., Wetzel, S. J., Panju, M. & Ganesh, V. Logic guided genetic algorithms (student abstract). *Proc. AAAI Conf. on Artif. Intell.* **35**, 15753–15754 (2021).

49. Błądek, I. & Krawiec, K. Solving symbolic regression problems with formal constraints. In *The Genetic and Evolutionary Computation Conference (GECCO '19), July 13–17, 2019, Prague, Czech Republic*, 977–984 (ACM, 2019).

50. Kubalík, J., Derner, E. & Babuška, R. Symbolic regression driven by training data and prior knowledge. In *Proceedings of the 2020 Genetic and Evolutionary Computation Conference*, 958–966 (2020).

51. Kubalík, J., Derner, E. & Babuška, R. Multi-objective symbolic regression for physics-aware dynamic modeling. *Expert. Syst. with Appl.* **182**, 115210 (2021).

52. Engle, M. R. & Sahinidis, N. V. Deterministic symbolic regression with derivative information: General methodology and application to equations of state. *AIChE J.* e17457 (2021).

53. Xu, J., Zhang, Z., Friedman, T., Liang, Y. & Broeck, G. A semantic loss function for deep learning with symbolic knowledge. In *International conference on machine learning*, 5502–5511 (PMLR, 2018).

54. Wang, W. & Pan, S. J. Integrating deep learning with logic fusion for information extraction. In *Proceedings of the AAAI Conference on Artificial Intelligence*, vol. 34, 9225–9232 (2020).

55. Cornelio, C., Stuehmer, J., Hu, S. X. & Hospedales, T. Learning where and when to reason in neuro-symbolic inference. In *International Conference on Learning Representations* (2023).

56. Li, T. & Srikumar, V. Augmenting neural networks with first-order logic. *arXiv:* **1906.06298** (2019).

57. Daniele, A. & Serafini, L. Neural networks enhancement with logical knowledge. *arXiv:* **2009.06087** (2020).

58. Xie, Y., Xu, Z., Kankanhalli, M. S., Meel, K. S. & Soh, H. Embedding symbolic knowledge into deep networks. *Adv. neural information processing systems* **32** (2019).

59. Li, T., Gupta, V., Mehta, M. & Srikumar, V. A logic-driven framework for consistency of neural models. *arXiv:* **1909.00126** (2019).

60. von Rueden, L. *et al.* Informed machine learning - a taxonomy and survey of integrating prior knowledge into learning systems. *IEEE Transactions on Knowl. Data Eng.* (2021).

61. Dash, T., Chitlangia, S., Ahuja, A. & Srinivasan, A. A review of some techniques for inclusion of domain-knowledge into deep neural networks. *Nature, Sci. Reports* **12, 1040** (2022).

62. Giunchiglia, E., Stoian, M. C. & Lukasiewicz, T. Deep learning with logical constraints. In *Proceedings of the Thirty-First International Joint Conference on Artificial Intelligence, IJCAI-22*, 5478–5485 (2022). Survey Track.

63. Sen, P., de Carvalho, B. W. S. R., Riegel, R. & Gray, A. G. Neuro-symbolic inductive logic programming with logical neural networks. *AAAI 22* (2022).

64. Evans, R. & Grefenstette, E. Learning explanatory rules from noisy data. *J. Artif. Intell. Res.* **61**, 1–64 (2018).

65. Sadeghian, A., Armandpour, M., Ding, P. & Wang, D. Z. Drum: End-to-end differentiable rule mining on knowledge graphs. In Wallach, H. *et al.* (eds.) *Advances in Neural Information Processing Systems*, vol. 32 (Curran Associates, Inc., 2019).

66. Law, M., Russo, A. & Broda, K. Inductive learning of answer set programs from noisy examples. *arXiv:* **1808.08441** (2018).

67. Nye, M., Solar-Lezama, A., Tenenbaum, J. & Lake, B. M. Learning compositional rules via neural program synthesis. In Larochelle, H., Ranzato, M., Hadsell, R., Balcan, M. & Lin, H. (eds.) *Advances in Neural Information Processing Systems*, vol. 33, 10832–10842 (Curran Associates, Inc., 2020).

68. Parisotto, E. *et al.* Neuro-symbolic program synthesis. *Int. Conf. on Learn. Represent.* (2017).

69. Valkov, L., Chaudhari, D., Srivastava, A., Sutton, C. & Chaudhuri, S. Houdini: Lifelong learning as program synthesis. *Adv. Neural Inf. Process. Syst.* **31** (2018).

70. Yang, F., Yang, Z. & Cohen, W. W. Differentiable learning of logical rules for knowledge base reasoning. *Adv. neural information processing systems* **30** (2017).

71. Cranmer, M. PySR: Fast & parallelized symbolic regression in Python/Julia. http://doi.org/10.5281/zenodo.4041459 (2020).

72. Cranmer, M. *et al.* Discovering symbolic models from deep learning with inductive biases. *NeurIPS 2020* (2020).

73. Branke, J., Deb, K., Dierolf, H. & Osswald, M. Finding knees in multi-objective optimization. *Parallel Probl. Solving from Nat. - PPSN VIII* 722–731 (2004).

74. `kneed` python library. https://pypi.org/project/kneed/. Version: 0.7.0.

75. Satopaa, V., Albrecht, J., Irwin, D. & Raghavan, B. Finding a "kneedle" in a haystack: Detecting knee points in system behavior. In *31st International Conference on Distributed Computing Systems Workshops*, 166–171 (2011).
